# Sustainability assessment of short food supply chains (SFSC): developing and testing a rapid assessment tool in one African and three European city regions

**DOI:** 10.1007/s10460-021-10288-w

**Published:** 2022-02-24

**Authors:** Alexandra Doernberg, Annette Piorr, Ingo Zasada, Dirk Wascher, Ulrich Schmutz

**Affiliations:** 1 Research Area 3 “Agricultural Landscape Systems”, Leibniz Centre for Agricultural Landscape Research (ZALF e. V.), 15375 Müncheberg, Germany; 2 DLR Projektträger, Deutsches Zentrum für Luft - und Raumfahrt e. V., 10178 Berlin, Germany; 3 SUSMETRO – Sustainable Design for Metropolitan Landscapes, 5038 EN Tilburg, The Netherlands; 4 Centre for Agroecology Water & Resilience (CAWR), Coventry University, Coventry, CV8 3LG UK

**Keywords:** Alternative food networks (AFN), Urban food system, Urban food policy, Tool development, Indicators

## Abstract

Recent literature demonstrates the contribution of short food supply chains (SFSC) to regional economies and sustainable food systems, and acknowledges their role as drivers for sustainable development. Moreover, different types of SFSC have been supported by urban food policies (UFP) over the few last years and actors from the food chain became part of new institutional settings for urban food policies. However, evidence from the sustainability impact assessment (SIA) of these SFSC in urban contexts is limited. Our paper presents an approach for the development of an SIA framework for different SFSC types. In addition, a practical application of the tool in four metropolitan regions (Berlin, London, Ljubljana and Nairobi) is tested. The conceptual development of the SIA framework is based on an in-depth analysis of existing SIA frameworks and methods for assessing the sustainability of agriculture, food chains and food systems and adapted to the specific needs of analysing SFSC in metropolitan regions. The operational value and utility of the framework and the tool were tested with social and natural scientists and local stakeholders. The assessment results demonstrate that SFSC seems to be more sustainable than the baseline (long global food chains) in the social dimension, but also feature specific strengths and weaknesses concerning their economic and environmental sustainability. This might give an indication for regional adjusted strategies and food chain innovations that improve the sustainability performance may be required. We found the tool useful for framing the dialogue between food chain actors, consumers and policy, because it makes benefits and trade-offs of the chain types operating in an urban–rural context more visible and communicable.

**Supplementary Information:**

The online version contains supplementary material available at 10.1007/s10460-021-10288-w.

## Introduction

### Sustainability of the food system and of food chains

Sustainable development and tools and indicators for assessing the current state and progress towards sustainability in the agri-food sector have had high relevance for research and policy agendas for many years (van Cauwenbergh et al. 2007; FAO 2013; Eakin et al. 2017; EEA 2019b).

A widespread negative perception of the sustainability and nutritional performance of the recent global, industrialized food system pointed to the need for technical, social or institutional innovations and possible alternative modes of food production, distribution and consumption enabling system change (Forssell and Lankoski 2015; Galli et al. 2015; Cagliano et al. 2016; EEA 2019a). Furthermore, these “alternative food systems, especially those that are developed with the aim to be more sustainable, offer particular lessons for wider agriculture, including how the sustainability assessment itself could be conducted” (Alrøe et al. 2016, chapter 4).

Academia and practice have contributed with many different paradigms and approaches on how to solve the problems in the current food system and establish more sustainable farming systems and food chains. They encompass, for example, technical and management innovations for conventional food systems framed in concepts such as sustainable intensification (Pretty 1997; Garnett and Godfray 2012; Loos et al. 2014; Weltin et al. 2018) or sustainable and integrated chain management (Seuring and Müller 2007; Carter und Easton 2011), and in alternative approaches for agricultural production and food systems, such as agroecology (Francis et al. 2003; IPES Food 2016), social innovations and new forms of food chains and governance, such as alternative food networks (AFN, Renting et al. 2003; Goodman et al. 2012) or food policy councils (Sonnino and Spayde 2014). The last two mentioned particularly reflect the observation that the current institutional arrangements of the ‘conventional’ food system seem to be inadequate to ensure sustainability (Michel-Villarreal et al. 2019). In addition, “the tension between ‘alternative’ and ‘conventional’ food mirrors that between ‘alternative’ and ‘mainstream’ approaches to sustainability” (Maxey 2007, p. 61).

The potential effects and perceived benefits of local food chains such as short food supply chains (SFSC; Marsden et al. 2002; Ilbery and Maye 2005; Galli and Brunori 2013) or alternative food networks (AFN, Renting et al. 2003; Freidberg and Goldstein 2011; Goodman et al. 2012) for balanced and sustainable urban and rural development has led to a growing interest among researchers, food activists, farmers, policymakers and planners worldwide (Jarosz 2008; Committee of the Regions 2011; European Union 2013; Kneafsey et al. 2013).

Cities are usually places of food consumption, distribution and retailing. By contrast, production, processing and packaging activities are only carried out to a small extent in the city and its immediate surroundings (Smit 2016, adapted from Ericksen 2008). As a growing number of people live in urban environments, the interrelationships between urban lifestyles and consumption patterns (urban diets) and the sustainability impacts of urban food systems (cities are mainly places of food consumption) on ecosystems, climate and societies (in urban and rural spaces) are becoming increasingly urgent (Seto and Ramankutty 2016; Landert et al. 2017). It is assumed that SFSC can contribute to multiple objectives of the United Nations Agenda for Sustainable Development (Ilieva 2017; UNIDO 2020). Moreover, different types of SFSC have been supported by urban food policies (UFP) over the last few years (Ilieva 2017) and actors from the food chain have become part of new institutional settings for UFP.

Against this background and in order to support informed decision-making of food system actors in city regions and justify policy interventions that promote SFSC chains and re-localization of food system knowledge about the sustainability impacts and adequate tools for measuring the impacts of these specific chain types, SFSC is required.

### Short food supply chains and their perceived sustainability impacts

An extensive body of literature over the last two decades has been studied on the occurrence, organisation and sustainability effects (benefits) of SFSC or AFN We use the more ‘technical’ term “short food supply chains” in the course of this study and focus on the way of food production, distribution and consumption including the organisational structure, spatial and social relationships between producer and consumer, and urban and rural spaces (Marsden et al. 2000; Renting et al. 2003; Aubry and Kebir 2013). This is to overcome the problems regarding the insufficient clarity and consistency in the usage of key concepts in this field of research and the conflation of the structural characteristics of alternative food chains with desired outcomes and/or actor behaviours (Tregear 2011). Herewith, we also change the attention “from ‘alternative’ food to ‘sustainable’ food” (Maxey 2007).

The SFSC are commonly considered as being more sustainable than global food chains and sustainability is even part of the definition (Jarosz 2008; Forssell and Lankoski 2015; Malak-Rawlikowska et al. 2019). It is stated that SFSC provide multiple benefits for the environment, society and individuals (consumers, farmers), for example, by reducing food miles and carbon emissions, fostering the economic viability of farmers and rural areas or changing eating habits with effects on public health (Ilbery and Maye 2005; Kneafsey et al. 2013; Mundler and Laughrea 2016; UNIDO 2020). Despite such direct sustainability impacts, it is also stated that they create indirect impacts related to learning and participation and creating awareness about sustainability-related issues in the food system, which is a very important aspect for the overall sustainability and food system transformation (Forssell and Lankoski 2015; Opitz et al. 2017).

Claims about the environmental, economic and social impacts of SFSC are numerous in the literature, but seldom proved by empirical research. Previous and recent studies often show a lack of systematic reviews and cross-county comparative case studies that investigate multiple dimensions of sustainability (Kneafsey et al. 2013; Michel-Villarreal et al. 2019). A few studies were published only recently that present comparisons of different chain types and across different countries with a huge amount of data gathered (Brunori et al. 2016; Malak-Rawlikowska et al. 2019; Majewski et al. 2020).

The majority of studies which support evidence are case studies from different regions and countries (mainly from the Global North) and are often based on qualitative estimations, representing the perceptions and experiences of food chain actors (Kneafsey et al. 2013). A lot of empirical studies focus only on one or a few specific types of SFSC and certain sustainability aspects, such as environmental sustainability (Forssell and Lankoski 2015; Mundler and Laughrea 2016; Michel-Villarreal et al. 2019) and a lack in the integration of the consumer perspective or sustainable consumption (Tregear 2011; Schader et al. 2014; Michel-Villareal et al. 2019). Another difficulty in this research field results from the limited access to qualitative and quantitative data of the benefits of SFSC (Mundler and Laughrea 2016) and a lack of baseline and longitudinal data (Kneafsey et al. 2013). Concerning the impacts of the agri-food sector and especially of local food systems (including SFSCs), it becomes obvious that the relevance and sufficiency of impacts were sometimes overestimated and possible counter-effects or trade-offs were neglected (Michel-Villarreal et al. 2019; Benedek et al. 2020).

Therefore, there is an ongoing discussion about the quality and coverage of the studies, the chain types assessed, the methods applied, and the impacts measured and their relevance (Tregear 2011; Kneafsey et al. 2013; Forssell and Lankoski 2015; Michel-Villarreal et al. 2019). The assessment of environmental sustainability, for example, is often operationalized by using concepts such as carbon footprints or food miles, which can be easily communicated to consumers (Pretty 1997; Hedberg 2016). On the other hand, the focus on carbon emissions and food miles “can divert attention from the far more fundamental and deep rooted social, economic and environmental changes that are required to tackle the sustainability challenge” (Coley et al. 2009, p. 931).

The main critique arises from the tendency in the public and scientific discussion to assume that SFSC or local food systems produce per se economically, socially and environmentally desirable outcomes and are, thus, inherently good or sustainable (Born and Purcell 2006; Tregear 2011; Michel-Villarreal et al. 2019). It is also not clear to date to which extent and how SFSC in general, but also specifically can address the sustainability objectives of city regions’ food systems better and how important the local conditions for the results are (Mundler and Laughrea 2016).

In our paper, we develop a conceptually and functionally novel approach to sustainability impact assessment (SIA) for SFSCs (SIA-SFSC) that meets the requirements of various actors in the food system, especially in a metropolitan context.

The SIA-SFSC is developed as a rapid assessment (quick scan) tool and attempts to fill the gap between quantitative data-based assessment of SFSC that address only one or two sustainability dimensions and single qualitative case studies that study one food chain type [e.g. community supported agriculture (CSA)] in one geographic region. We also identified the need to adopt more holistic research approaches that allow the evaluation of trade-offs and balance among the social, economic and environmental sustainability for different SFSC.

The ambition of this research paper is twofold: (1) we want to contribute to a more differentiated assessment framework for SFSC types and create awareness about potential trade-offs and the strengths and weaknesses of the diverse SFSC types. (2) We test whether this approach can be implemented as a tool that is sensitive to different sustainability contributions of different SFSC types in various regional contexts and if such a tool could be used for benchmarking.

The paper is structured as follows: in the following chapter, we provide a literature review about existing approaches that assess the sustainability of farms, food chains and food systems and focus especially on approaches to SFSC and local food systems. The third chapter describes the SIA tool development procedure in detail. Selected results are presented in chapter four. Finally, we discuss the feasibility of SIA-SFSC for SFSC analysis.

## Literature review on sustainability impact assessment tools in the agri-food sector

### Types and variation of tools

A plethora of concepts, methods and indicator sets exist for sustainability assessments generally and for farms, food chains or food systems specifically in the academic literature. Farm-level assessment tools especially gained a lot of attention, and were studied and applied by many researchers (Binder 2010; Marchand et al. 2014; Schader et al. 2014; de Olde et al. 2016). The reviews and comparisons by van Cauwenbergh et al. (2007), Gasparatos and Scolobig (2012), Singh (2012), Schader et al. (2014), Prosperi et al. (2015), meta-reviews by Sala et al. (2015) or categorization schemes by Ness et al. (2007) and the FAO (2013) provide some guidance about and overview of tools and indicators.

Sustainability assessment frameworks and tools were developed for different purposes, scopes and subjects of investigation and show different degrees of complexity. They also differ in their degree of stakeholder involvement and requirements regarding data and knowledge stocks in the implementation phase (Ness et al. 2007; Binder 2010; Marchand et al. 2014; Alrøe et al. 2017). The SIA tools were usually characterized according to their purpose, the level of assessment (unit of investigation), geographical scope (local, national, global), sectorial scope (applicable to all, specific agricultural, food products or farm types), thematic scope (dimensions of sustainability: environmental, economic, social or integrated) (Schader et al. 2014).

**Fig. 1:**
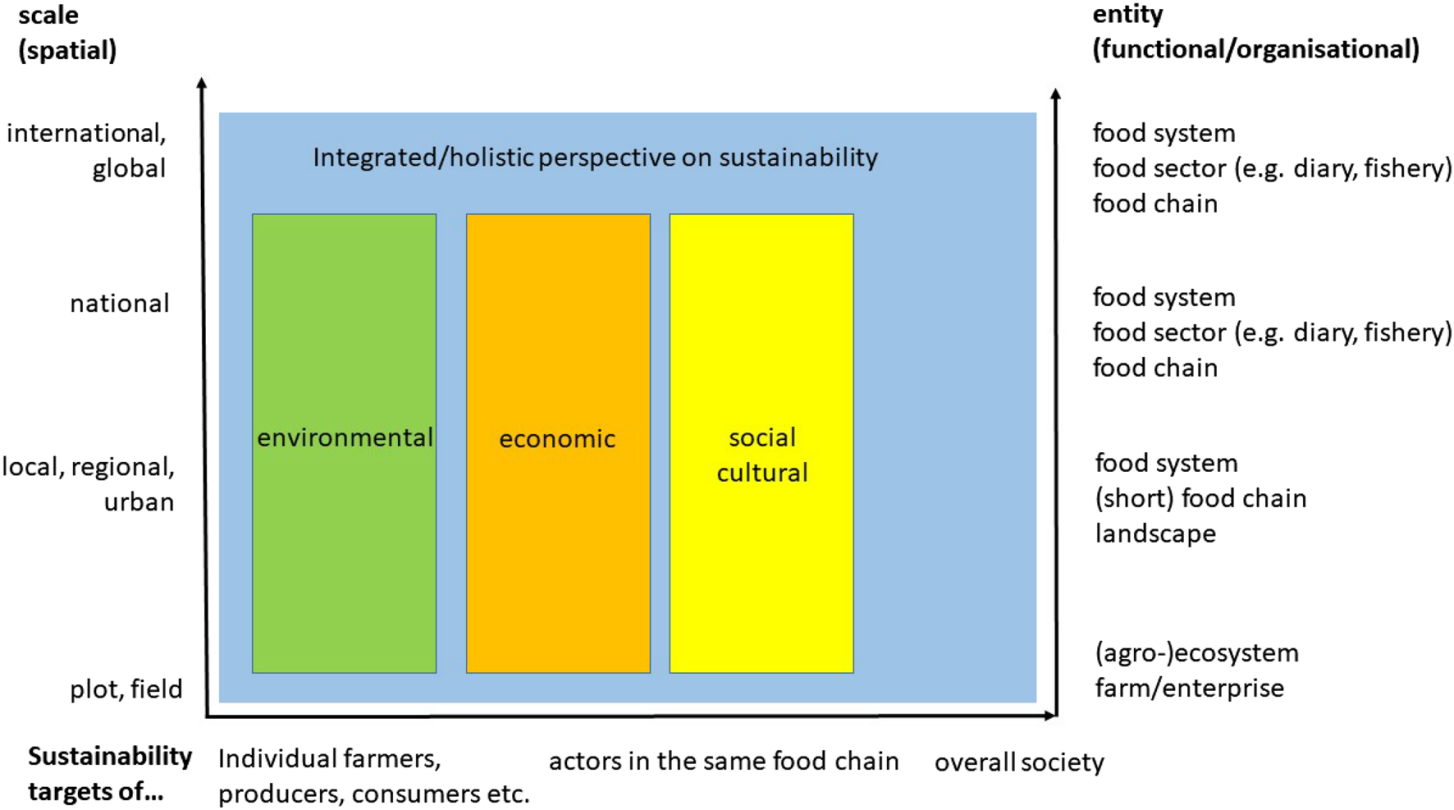
Scope, scale and sustainability dimensions of assessments in the agri-food system. Source: Own compilation based on Sauvenier et al. (2005), Peano et al. (2014) and Schader et al. (2014)

Assessment tools in the agri-food sector focus on either the agricultural production at farms, including different farming or production systems, such as organic vs. non-organic agriculture (Lillywhite et al. 2012; Schader et al. 2012), urban rooftop farming (Sanyé-Mengual et al. 2015), or single stages within the food chain (e.g. processing, transport). The units of assessment range from a single agricultural commodity to commodity groups (such as meat), firm level or food sectors (e.g. diary, agriculture, fishery) level to an entire food system (Prosperi et al. 2015). Tools can cover spatial scales from single fields to local or global food chains, and include sustainability targets of different stakeholders ranging from individuals to the whole society (Fig. 1).

In addition to Schader et al. (2014), other researchers categorize tools according to their temporal characteristics and assign whether a tool evaluates a past development (ex-post), describes a current state or performance of a food chain or food system, if it used for assessing future (desired) outcomes from policy changes or improvements in a production process (ex-ante, change oriented) or even addresses questions of inter- and intragenerational equity (Ness et al. 2007; Alrøe et al. 2017; Landert et al. 2017). Another useful differentiation of assessment tools was made, for example, in the review by Binder (2010), who categorized three types of assessment methods according to the degree of stakeholder involvement: (1) top-down farm assessment methods, (2) top-down regional assessment methods with some stakeholder participation, and (3) bottom-up integrated participatory or transdisciplinary methods with stakeholder participation throughout the process.

The perceptions of sustainability of different stakeholder groups (e.g. farmers, consumers, policymakers) or the whole society are relevant for defining a target system or reference frame for the assessment (Kloppenburg et al. 2000). Furthermore, Smith (2008) and Caracciolo et al. (2011) assigned typical responsibilities to different actor groups for the sustainability of a food chain.

The tools were usually designed for multiple purposes, which include research, monitoring, (self-)assessment and benchmarking, advice (farm, policy), certification, consumer information and landscape planning (Marchand et al. 2014; Schader et al. 2014). They fulfil different functions, such as information and communication, facilitate learning and the exchange of ideas, and knowledge or support decision-making and planning. The use in practice can also be irrespective of the original intention of the tool developer and many tools were used for more than one purpose (Marchand et al. 2014; Peano et al. 2014; Schader et al. 2014).

### Tools and frameworks for SFSC and local food systems and criteria for tool selection

A lot of frameworks, tools and indicator sets have been developed during the last few years for assessing the sustainability of farms, the agricultural sector and food chains. Assessment frameworks and indicator sets for food systems (DEFRA 2011; Prosperi et al. 2015) and specifically for urban food systems and urban food governance have come up in science and practise more recently (Landert et al. 2017; FAO 2019).

We give a brief overview in the following about assessment tools that address three or more dimensions of sustainability and allow the evaluation of economic, environmental and social aspects in food chains or food systems (Table 5 in the supplement). The approaches and tools presented were chosen in order to exemplify the broad variety of methods and scopes and the information does not claim to be complete.

Some of the frameworks are well-established and several empirical studies exist which applied these approaches in the context of food, for example, Monitoring Tool for Integrated Farm Sustainability/MOTIFS, (Meul et al. 2008), Sustainability Assessment of Food and Agriculture Systems/SAFA (FAO 2013) and Social Return on Investments/SROI (SROI Network 2012). Many of them are very data-intensive (mostly quantitative primary and secondary data) and require very specialized scientific or technical knowledge, whereas other tools use the knowledge of practitioners or other data sources (e.g. SROI), or combinations of existing statistical/accounting data and experts or stakeholders estimations (e.g. MOTIFS, Public Goods Tool/PG: Gerrard et al. 2011, 2012; FAO 2019).

Assessment approaches to environmental sustainability are often expert-based and apply complex methods, such as carbon accounting and footprint analysis (Coley et al. 2009). Life cycle assessments (LCA) are especially widely used for assessing food supply chains of all kinds (Hagelaar and van der Vorst 2002; Roy et al. 2009), including SFSC (Kulak et al. 2015; Brunori et al. 2016) or urban agriculture (Sanyé-Mengual et al. 2015). Assessment tools for farms are widespread, but they usually do not assess impacts on soil quality, biodiversity or ecosystem services and neglect social, health or ethical issues of food production (Schader et al. 2012, 2014; MacPherson et al. 2020). Social Life Cycle Assessment (S-LCA) tackling these social issues were evolved later than environmental LCA, but “the level of methodological development, application, and harmonisation of Social LCA is still in a preliminary stage” (Sala et al. 2015, p. 2). Exemplarily, the PG Tool and the SAFA Guidelines (FAO 2013), which provide one the most holistic assessment approaches for farms and beyond, have a broader coverage of environmental issues.

Although there have been many attempts to assess the sustainability of SFSC (for overviews, see, for example, Kneafsey et al. 2013; Brunori et al. 2016; Michel-Villarreal et al. 2019), we found only a few examples of assessment approaches that have been applied specifically to SFSC and explicitly in the urban context (e.g. SAFA; Landert et al. 2017; Malak-Rawlikowska et al. 2019).

The concepts and framings of the sustainability of food chains and systems are usually based on reflections or the knowledge of specialists in science and policy (Kloppenburg et al. 2000). Only a few approaches attempt to include producers and consumers and apply participatory processes in the tool development (Kloppenburg et al. 2000). Integrated participatory and transdisciplinary approaches have been investigated in recent years (e.g. Landert et al. 2017; Sanyé-Mengual et al. 2018), but the experiences with these participatory sustainability assessments were divergent in practice (Triste et al. 2014).

In contrast to the growing number of assessment tools and frameworks, there is limited guidance and criteria concerning how to choose the appropriate tool. Therefore, the selection is based either on stakeholders’ needs or pragmatic considerations, such as data availability (de Ridder 2007; Gasparatos and Scolobig 2012; Marchand et al. 2014). As a consequence of the specific strengths and weaknesses of the different SIA approaches and tools, scholars in this research field conclude that the approaches need be tailored for a specific purpose considering the specific question or problem and scope (de Ridder 2007; Schader et al. 2014). Another option is to combine different tools, methods and indicators in a modular way or to use them complementarily (Marchand et al. 2014).

**Fig. 2:**
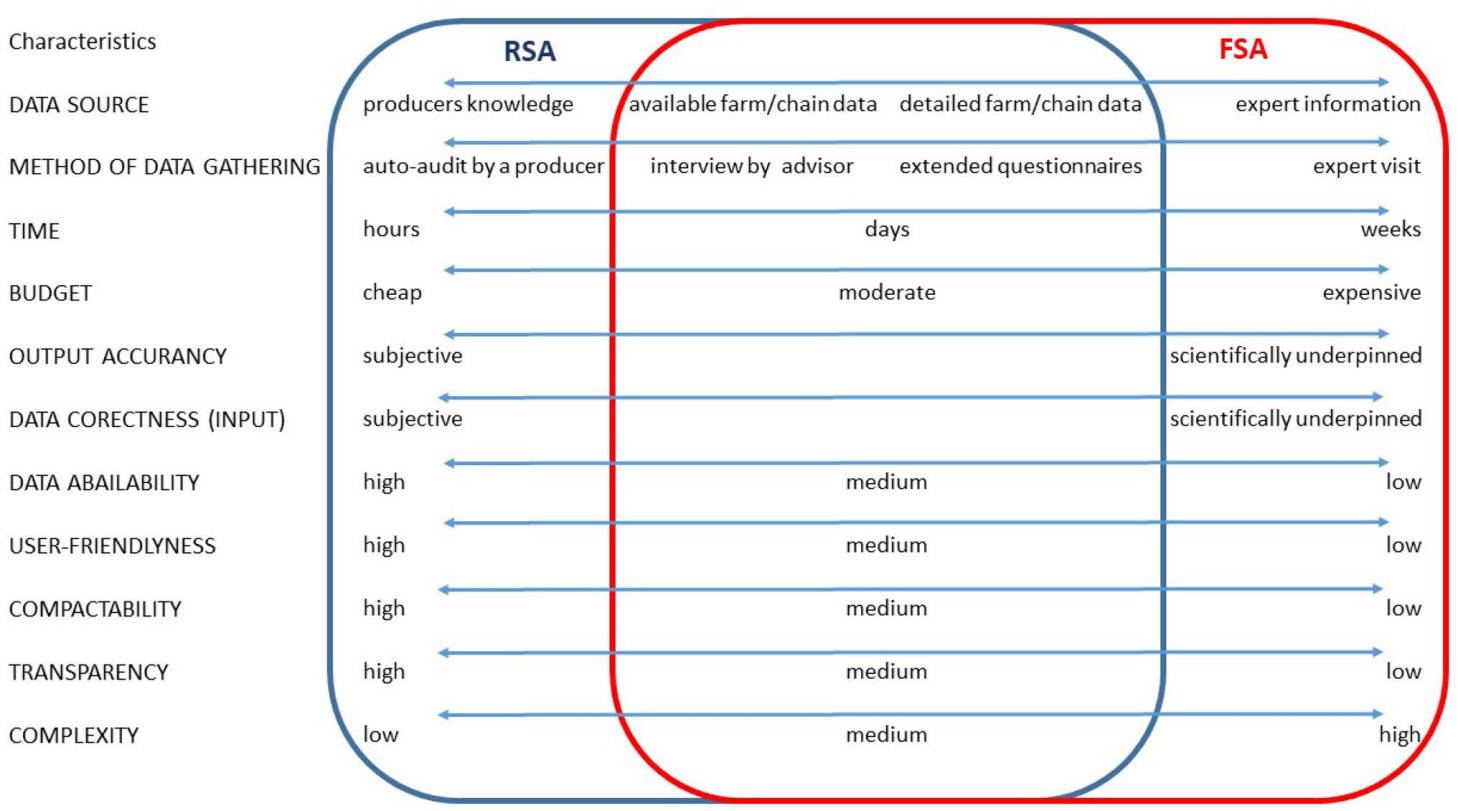
Main characteristics of full and rapid sustainability assessment tools for farms and food chains. Source: Own illustration adapted from Marchand et al. (2014)

Some guidance for tool selection can be drawn also from the differentiation between Full Sustainability Assessment (FSA) and Rapid Sustainability Assessment (RSA) tools by Marchand et al. 2014 (Fig. 2). This framework was developed to compare two types of indicator-based assessment tools for agriculture (farms), but is also applicable for other tools with a different scope. FSA and RSA vary mainly in their applicability (e.g. time, budget, method of data gathering), data requirements and certain quality criteria, such as user-friendliness, transparency, complexity and output accuracy. Tools such as rapid assessment are oriented towards learning and communication, can help to raise awareness and highlight areas with good or bad performance and can, therefore, support the identification of areas for action (Marchand et al. 2014).

## Methods and tool development procedure

We decided to design our own rapid assessment (quick scan) tool to reflect the state-of-the-art and anticipate ongoing developments in this field. The process of developing a tool for assessing urban (short) food supply chains included three main methodological steps. The first step was to specify the tool requirements and define the system boundaries (food chains). We developed a typology of generic SFSC types (2A) and defined impacts areas and indicators (2B) in the second step (tool development). The third and last step encompassed the tool testing and implementation.

### Defining tool requirements and system boundaries (Step 1)

We aimed to develop our own rapid assessment (quick scan) tool within the FOODMETRES project which covers the following functions: benchmarking, decision-making, information and communication in different regional settings, i.e. the political, social and economic conditions, in which (1) the tool is applied (experiences, literacy level of the participants) and (2) the chains were assessed (city regions in Europe and Africa). The SIA-SFSC should encompass the environmental, economic and socio-cultural dimension of sustainability and integrate impacts on both producers and consumers. An adoption of the indicator framework towards sustainability targets of cities and regions (urban food policy and planning) or food chain actors (e.g. for self-commitments or reporting requirements) should be possible. The benchmarking refers to the comparison of the sustainability performance of food chain types across city regions. As a tool of information and communication, it should be able to identify and communicate strengths and weaknesses as well as trade-offs of different food chain types in terms of its contribution to sustainability. The information can be used to support decision-making and the setting of priorities in policy (e.g. which food chain types address which sustainability target best?) or support municipal food system assessments. It can be used to identify potential for improvements (innovation goals), which is relevant for the food chain actors in SFSC. This kind of information can also support communication (e.g. promotion of specific chain types) according to specific regional needs and societal challenges.

The system boundaries of the SIA-SFSC are defined based on the food chain (as a unit of investigation; Brunori et al. 2016). We included the stages food production (farm level) to consumption (consumer household), including off-stream activities, such as transport and packaging, and excluded upstream activities of input suppliers (e.g. fertilizer, machinery). The advantages of the food chain level as a unit of analysis for sustainability assessments is that it provides an appropriate unit “that can be adopted by practitioners and researchers to assess the interplay between economic and ecological dynamics” (Boons et al. 2012, p. 141) and offers, at the same time, an arena where actors are able to co-ordinate and govern their actions (Boons et al. 2012). The SFSC are characterized by a very limited number of intermediaries “from farm to fork” and the supply of usually fresh, seasonal food with a low processing level. The different regional and SFSC types were compared against global (long-distance), complex food chains with many intermediaries based on large-scale industrialized agriculture (baseline).

Regarding the SIA-SFSC, we were looking for a holistic approach to compare different conceptions of food chains in metropolitan regions with each other and address the specifics of SFSC types, such as the close consumer-producer relationship, in which the consumer takes over a more active role in the food chain (Mundler and Laughrea 2016). These chains usually involve small-scale producers and, to some extent, consumers (e.g. CSA), which operate in different institutional settings, for example, associations or informal collaborations. These two particularities cause limitations in data availability, respectively, data coverage of these specific food chains. Another difference to the assessment of long global food chains is that they often focus on the comparison of different production systems or the mode of transport as well as different commodities (process and product-oriented approaches), but seldom compare different types of chain organisation (food chain types) or assess impacts on consumers.

### Tool development (Step 2)

#### Defining short food supply chain types (Step 2A)

As the SIA-SFSC should be carried out for different types of SFSC, it was necessary to develop a consistent set of SFSC used in international studies and to be found in the FOODMETRES project case study cities.

We found various definitions of an SFSC with a ‘common set’ of characteristics within the agri-food studies and policy documents about SFSC investigated (e.g. Marsden et al. 2000; Renting et al. 2003, 2012; Goodman 2004; Watts et al. 2005; Jarosz 2008; Committee of the Regions 2011; Tregear 2011; Galli and Brunori 2013; Kneafsey et al. 2013; European Union 2013) which include:

- Spatial proximity: shortening the total distance (in physical distance and time)
- Social proximity: personal interaction of producer and consumer (face-to-face)
- Limited number of intermediaries (zero or one)
- Goal (e.g. more added value for farmers and producers, local economic development)
- Changing food system processes by re-embedding, reconnecting, re-localizing and re-socializing

While the first three criteria can be seen as a minimum definition, the last two are extended definitions for SFSC. As SFSC are embedded in a territorial and social context, a variety of ways exist regarding how regional food is produced, processed and distributed in supply chains. Therefore, we chose an approach which puts the consumer-producer relationship into focus. This approach is oriented on the definition of the Committee of the Regions (2011).

Concerning the SIA of SFSC, we differentiate, firstly, between very short and rather short food chains considering the number of intermediaries between producer and consumer (zero, respectively, one) and, secondly, categorize the market relation between consumer and producer and, thirdly, relate them to commercial transactions schemes.

The market relation include four categories:

- Consumers as producers (transaction scheme: not existing)
- Producer‐consumer partnerships (transaction scheme: business‐to‐consumer)
- Producer direct sales to consumer (transaction scheme: business‐to‐consumer)
- Producer direct sales to intermediates/no direct consumer‐producer relation (transaction scheme: business‐to‐business and business-to-administration).

These four main categories for chain types can be further differentiated according to the kind of intermediate chain actors (retail, hospitality industry, public procurement).

Based on these distinctions, we specify eight main types of SFSC and related subtypes and venues. The point of sale can be urban, peri‐urban or rural (Table 1).

**Table 1:**
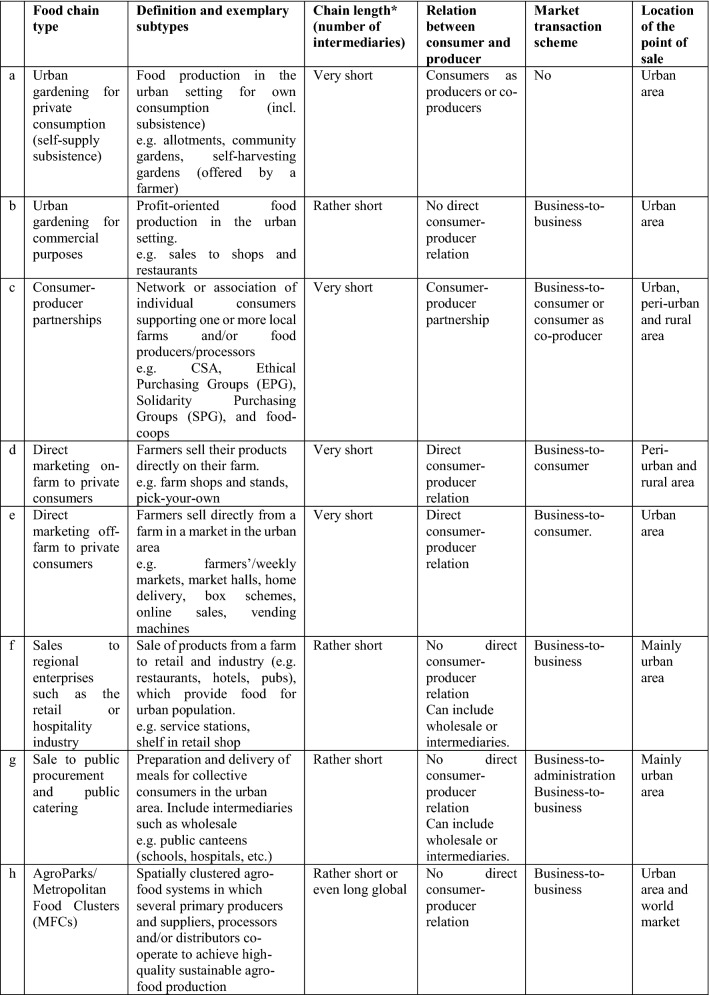
Description of the eight short food supply chain types

Source: Own compilation based on literature

^*^Chain length: very short-usually no intermediaries, rather short-one or more intermediaries

It is important to note the distinction between SFSC and local or urban food systems. An urban food system “includes all processes that food passes through, from its production over processing, transportation, retail, consumption to disposal of kitchen and table waste (incl. food waste) as well as all actors and institutions that influence these processes” (RUAF n.d. based on Wiskerke 2009). A food system also includes inputs and outputs of food production and consumption as well as resources, actors and governance in a defined geographic area (Kneafsey et al. 2013; Blay-Palmer et al. 2018), whereas SFSC focus on the reduced number of intermediaries. We consider SFSC in our assessment study as one element of an urban food system which organises the way from food production to food consumption within a metropolitan region and with a limited number of intermediaries.

#### Defining sustainability, impact areas and indicator set (Step 2B)

Following Schader et al. (2014), we had to determine initially what sustainability of food chains in the given context (whether from a farm, chain or society perspective) means. We adopted the most common framework for defining the sustainability dimensions, which consists of three main dimensions (environmental, social and economic) and in which all three elements are satisfied simultaneously (Maxey 2007; UN 2015). We applied a normative approach to the SIA-SFSC to focus on their perceived contribution to sustainable development and policy goals and decided to apply a benchmarking method to assess the different food supply chains regarding the maximum benefits. The objective was to compare different generic chain types regarding their contribution to these goals in a normative sense (for a more detailed description see Zasada et al. 2014).

The impact areas and set of indicators were then selected based on an extensive review of the existing research literature and case studies. We focused here specifically on (1) general literature dealing with sustainability impacts on assessment approaches, (2) specific literature about the sustainability of local or SFSC and collected information about the impact fields used.

We found from the literature that despite varying priority settings in the different assessment approaches, there is a relatively broad consensus about the relevant sustainability issues of SFSC (normative assumptions or sustainability attributes) which leads to some overlapping sustainability indicators (which were used more frequently in empirical studies). Regarding the environmental dimension, they include (efficient) resource use, impacts on environment and landscape, and the length of the food chain (e.g. transport distance) and, therewith, reduction of greenhouse gases are frequently considered. The economic dimension usually encompasses added value, growth and competitiveness, logistics efficiency and rural development. Regarding the social and cultural dimension, employment, food security and safety, and the local community embeddedness are studied (Zasada et al. 2014).

We defined a set with five indicators for each of the three sustainability dimensions in an iterative process with project partners, external experts and local stakeholders. A preliminary indicator set (see Table 7 in the supplement) and typology of food chains was pretested with 14 scientists from the FOODMETRES consortium (covering Germany, Great Britain, Italy, Kenya, Slovenia and The Netherlands) and subsequently adapted (for a detailed description of the methodological development, see Zasada et al. 2014). The final list with 15 indicators applied in the case study cities is presented in Table 2. It is important to note that the impact areas address different scales within the urban food system: a) impacts related to individual food chains (actors), such as food security or income, and b) impacts related to the overall regional food system (urban–rural society), such as (agro-)biodiversity. Furthermore, they can be linked to innovation goals in SFSC or political agendas (Wascher et al. 2015).

**Table 2:**
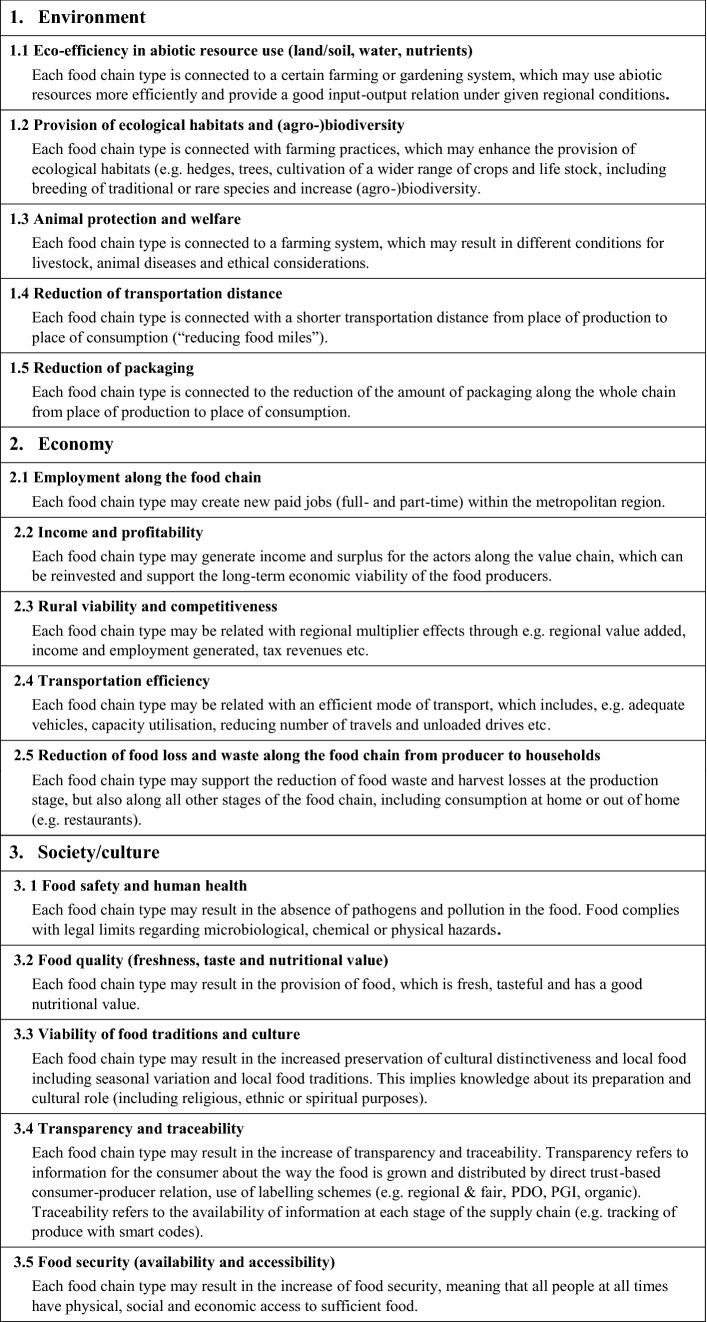
Final list of indicators

Source: Own compilation

### Testing and applying the SIA-SFSC (Step 3)

#### Pretest

A preliminary SIA-SFSC tool was tested in a first step within the project team (14 persons) making use of the existing expert knowledge from the interdisciplinary team. The pretest aimed at technical and logical feasibility and tested the comprehensiveness of the questions and the feasibility of the chain types and impact areas. Partner scientists were also asked to evaluate the relevance and feasibility of the impact areas. As a result of the pretest, two chain types and a few impact areas were revised (see Table 6 in the supplement) and implemented for the online expert survey.

#### Expert-based assessment (online survey)

In a second step, the SIA-SFSC tool was tested with international experts using an online survey applying the software package SoSci Survey (https://www.soscisurvey.de/). We conducted a structured research (for a detailed procedure description see Zasada et al. 2014) and identified and contacted 176 experts in Europe, of whom 37 responded and gave assessments for seven generic chain types, which included: urban gardening for private consumption (subsistence), urban gardening for commercial purposes, consumer-producer partnerships, direct marketing on-farm to the private consumer, direct marketing off-farm to the private consumer, sales to regional enterprises, such as the retail or hospitality industry, AgroParks/Metropolitan Food Clusters (MFCs). In addition to questions about the background of the respondent (field of expertise, country of origin), the survey consists of 15 closed-ended questions, each focusing on one sustainability impact area.

#### Participatory assessment in case study city regions

In the third step, the tool was applied to four of the six case study cities of the FOODMETRES project and for selected SFSC with significance for the respective city regions in Europe (Berlin, Ljubljana, London) and Africa (Nairobi). Accordingly, one or more local workshops with local experts and stakeholders were organised in the cities in 2014 (Table 3). The participants were contacted according to their experience with food chains and specifically for the SFSC types studied (e.g. urban agriculture, direct marketing) and included consumer and producers sides as well as NGOs and officials. It was ensured that the stakeholders represented different actor groups. The chains assessed also included regional subtypes, such as urban family subsistence farming (Ljubljana), which is common for many post-socialist and African countries or community-based urban agriculture in the case of Nairobi.

**Table 3:**
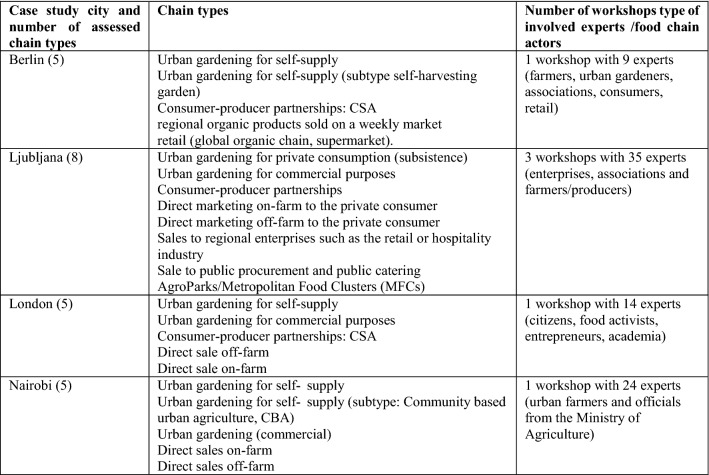
Overview about the chain types assessed in the pilot and the four case study cities

Source: Own compilation

All participants were introduced to the project objectives and the SIA procedure. The preparation and implementation phase was more intensive in Nairobi because many of the urban farmers within the group of participants invited (officials from the Ministry of Agriculture and urban farmers) had a low literacy level and needed support from students guiding them through the workshop and filling in the assessment form. The assessment in all workshops is based on previous knowledge, experiences and self-evaluations of the participants from various fields/stages in the food chain, who evaluated the specific SFSC against the baseline.

## Results

### Selected results from the impact assessment from four city regions

In the following, we present selected results from tool application in the four case study cities mentioned. The results from the online survey and detailed results from the workshops and other commodity groups are provided in the supplementary information (Figs. 4–18).

The results from the SIA-SFSC were displayed in two different forms, which can be easily understood by the different chain actors: (1) we used radar diagrams for the sustainability profile of the different chain types. This representation allows the identification of the specific strengths and weaknesses of each chain type, fields for improvement and sustainable chain innovations. (2) The ranking scheme displays overall sustainability performance of the food chains in the cities studied and shows where the highest sustainability impacts can be expected in the urban context given and, therefore, might be favourable for achieving sustainability targets of the city.

**Fig. 3:**
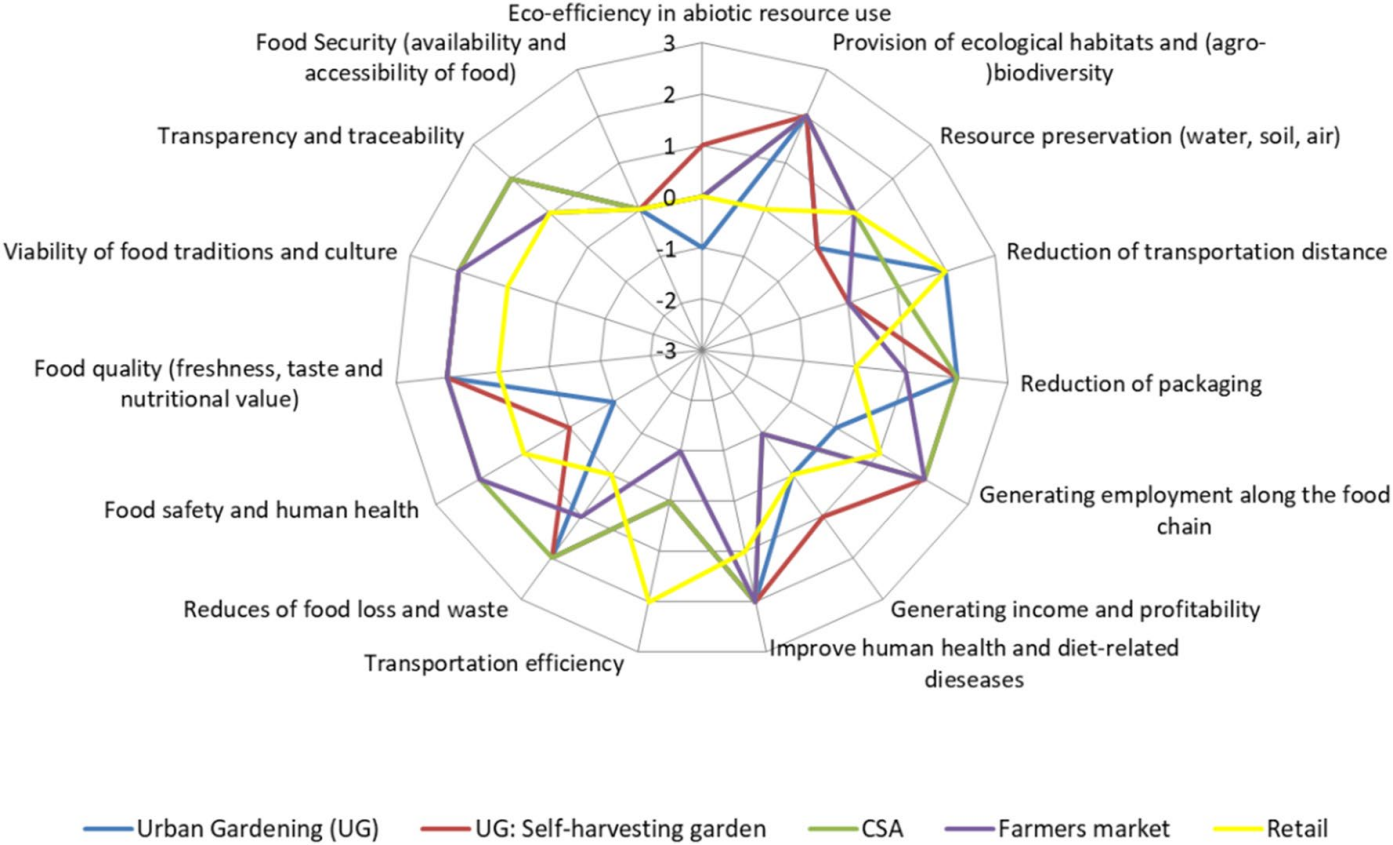
Results presentation of Berlin pilot for organic food (SFSC versus mass distribution in retail, 2nd workshop round, scale: + 2 = very positive to − 2 = very negative, 0 = no impact). Source: Own illustration

The results from the impact assessment with stakeholders show that the different food chain types (very short and rather short) account for mainly positive impacts in comparison with the global, long food chains and feature quite specific sustainability performance profiles (Table 4, Figs. 4–18 in the supplement). These positive impacts were also perceived for organic food in the case study city of Berlin (Fig. 3). Here the baseline scenario was an organic long global food chain with distribution via supermarkets (discounter) for vegetables versus urban gardening, self-harvesting garden (urban gardening), CSA, off-farm selling in a farmers’ market and regional retail.

**Table 4:**
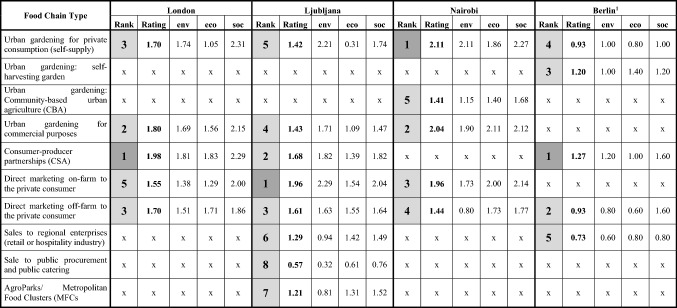
Rank and rating (average values) across all sustainability dimensions for the chain types assessed (Rank 1 = highest average value among the chains in the case study city)

Source: Own compilation based on workshop results, Berlin (pilot): N = 9; London: N = 16; Ljubljana: N = 17; Nairobi: Urban gardening (self‐supply): N = 21; Urban gardening (commercial): N = 22; Direct sales on-farm: N = 24; Direct sales off-farm N = 19; Community‐based urban agriculture: N = 20

^1^Berlin: scale + 2 = very positive to − 2 = very negative, 0 = no impact, other cities: very negative (− 3) to very positive (+ 3), 0 = no impact

The results from Berlin show that the five examples of regional supply considered can contribute in varying degrees to a higher sustainability. However, their major strength is in the social dimension, where the highest scores were given by the local stakeholders. The qualitative assessment from the Berlin workshop detected weaknesses in comparison to the reference system mainly in three areas of impact: (1) resource efficiency, (2) transportation efficiency and (3) food security (urban agriculture initiatives). Additionally, income and profitability were assessed quite low or even negatively by the stakeholders (Fig. 3), who explained this by the low income and wage level in German agriculture.

There is also consensus among the stakeholders involved in the other three case study cities (London, Ljubljana and Nairobi) on positive impacts of SFSC (mean score mostly well above 1, sometimes up to above 2, Table 4, Figs. 4–18 in the supplement). The majority of the SFSC types studied address social aspects before and, to a lesser extent, environmental and economic objectives. We assume that these social aspects (especially transparency, traceability and food quality) reflect mainly the needs and preferences of (individual) consumers and society objectives rather than the situation of the food producers.

In terms of environmental sustainability, the fields with the highest impacts are eco‐efficiency in abiotic resource use and a reduction of transportation distance. Interestingly, the workshop participants differentiated between the positive impacts by a reduced transportation distance (high impacts in the case of urban agriculture) and the rather lower transportation efficiency of SFSC associated with small-scale producers or consumer journeys (e.g. sales on-farm).

Economic-related impacts generally received low ratings, except in Nairobi. Economic sustainability in terms of income and profitability are mainly provided by chain types with direct consumer contact, such as CSA, sales on- and off-farm to consumers or commercial partners (retail or hospitality industry). The different forms of urban gardening do not perform so well and have mainly low or even negative impacts in contrast to the other chain types (Berlin, London, Ljubljana). The evaluation by the workshop participants for Nairobi was a bit different. Here, urban gardening for self-supply and commercial purposes ranked better than the other chain types studied.

The best performance across all sustainability dimensions seems to feature very SFSC types with direct consumer‐producer interaction, such as CSA and direct sales on‐farm (European cities) and different forms of urban food production in the case of the African city studied. Though there is only limited practical experience, also regarding the concept of MFCs/AgroParks, positive impacts of the overall sustainability were expected from the workshop participants (Table 4).

The expert assessments from the online survey tend to show fairly similar results (Figs. 4–18 in the supplement), although there are significant differences in the degree of perceived contribution of SFSC to sustainable food supply in metropolitan areas. Whereas neutral perspectives prevail, at least, among academic experts, the participants in the regional workshops have a significantly more positive perspective towards SFSCs in comparison to long global food chains. This is hardly surprising as there are, to a large extent, activists and stakeholders directly involved in regional food supply, having close insights and sharing more direct experiences with the effects of SFSC.

Summing up, the results demonstrate that not all sustainability objectives can currently be achieved at the same time and trade-offs probably exist. Each chain type shows specific strengths and weaknesses. Nonetheless, according to the qualitative assessment of local stakeholders and international experts, the SFSC investigated mostly perform better than the baseline (long global food chains).

### Evaluation of the SIA-SFSC tool

The SIA was conducted for chain types without a regional context (online survey) and for chains in their specific regional setting (SIA workshops in four case study regions). The chain typology was feasible and could be adapted to regional conditions (e.g. family subsistence farming in Slovenia or community-based urban agriculture in Nairobi) and other commodity groups (pork, fruit). The SIA procedure was very useful for a structured discussion about the sustainability of food chains, but needed quite some time for information and explanation about the chain types and impact fields in almost all case studies depending on the previous knowledge and expertise of the stakeholders invited to participate. Nonetheless, t was possible to conduct the assessment within few a hours or half a day (rapid assessment) in all workshops.

It generally occurs that participants in the regional workshops have a significantly more positive perspective towards SFSC in comparison to long global food chains than the experts in the online survey. This is hardly surprising, as the former are, to a large extent, activists and stakeholders directly involved in regional food supply, having close insights and sharing more direct experiences with the effects of SFSC, whereas neutral perspectives prevail among academic experts.

Differences do not exist only between expert opinions and those of regional food stakeholders and activists, but also across the regional workshops. The SFSC are considered to contribute positively to sustainable food supply throughout all impact areas, especially in the Nairobi case study. Economic categories, such as employment, income generation and economic viability, and social aspects, such as food safety and security, are particularly ranked substantially higher than in the other regional case studies and expert survey. By contrast, the participants from the Berlin workshop had problems to assess food security, which was ranked quite low, similar to that in Ljubljana. In London, however, food security was one of the most relevant impact fields (rank 3). Since larger differences between the urban food systems in Kenya and the European countries existed, this would lead to another target system for sustainability and, therefore, to another set of relevant impact fields.

Nevertheless, the SIA also had some weaknesses which evoked some criticism from the participants of the online survey and the workshops. Critical feedback from the online survey concerned the questions how the different chains types are organised in practice (e.g. intensity of production, chain organisation) and their regional setting, which might lead to different evaluations. A second area of critique was the relationship between the actual impact and the potential or future impact on the food system. In the workshops, some of the urban gardeners (London, Ljubljana) had problems with understanding the food chain terminology and other participants reviewed the assessment procedure as rather top-down and scientific. This shows the challenges of different degrees of stakeholder involvement in tool development and implementation.

## Discussion

In this study, we created a typology of SFSC and developed and tested a tool that allows us to compare the perception of sustainability impacts of different chain types that can be found in metropolitan regions. The tool worked out in the different socio-economic and natural contexts of four case study cities in Europe and Africa and with different actor groups (e.g. farmers, food initiatives, consumers, public administration). In contrast to many other assessment tools and frameworks available, the SIA-SFSC includes three dimensions of sustainability in addition to the producer and consumer perspective. Aspects of the production and distribution were considered as well as the wider society. The social dimension addresses more or less the consumer or societal perspective.

The chain typology developed expands existing ones (Pretty 2001; Renting 2003) by differentiating between very short and rather short local food chains, which is seen as useful for studying the impacts of local food chains (see also Malak-Rawlikowska et al. 2019). In addition, we defined transactions schemes which create links to the ‘main stream’ food chain research and assigned the point of sale (urban, peri-urban, rural space). This might be useful for city regions’ food policy and planning and existing funding schemes. The chain typology was useful to cover the heterogeneity of SFSC (Vittersø et al. 2019) and demonstrates that each chain type shows quite clear differences in their sustainability performance. This allows a benchmarking and can support the decision-making of food chain actors and policymakers dealing with UFP. In addition, the distinct chain differentiation can be used in academia for empirical studies about SFSC. A possible extension could be to assign certain stages in the chain and impact areas to the responsibility of specific chain actors (Smith 2008; Peano et al. 2014).

All in all, the SFSC types, assessed in the four case study cities, account mainly for positive impacts in comparison with the long global food supply chains. They were generally associated particularly with benefits for society and the environment. Similar results were presented in the empirical study of Mundler and Laughrea (2016), which found mainly positive (farmers’ welfare, local development, welfare of the community and environmental protection) or neutral effects (economic role of SFSC in local economy, the access to fresh and healthy food). The socio-economic benefits and shortcomings for the producers were, for example, studied by Schmutz et al. (2017), Opitz et al. (2019) Benedek et al. (2020) and Malak-Rawlikowska (2019). The latter study reveals a slight preference for SFSC (in general) and against long chains according to the better prices, and regular and assured payments (economic stability), but also certain shortcomings of specific SFSC types, such as pick-your-own and internet sales, due to small quantities and the occasional character of these purchases. Long channels, on the other hand, provide the chance to sell larger quantities and arrange long-term contracts. An obvious drawback from these channels is the lower bargaining power of producers in the long chains and especially for sales to intermediaries.

The results from the Nairobi case study need to been seen against the background of the specific regional socio‐economic situation in the Kenyan capital and other African cities. Here, concepts of (‘traditional’) regional and short food supply (formal and informal) play a more serious role in addressing issues such as food security and employment, especially for the urban poor, whereas supermarkets connected with longer chains and international products are the source of supply for the middle class (Zasada et al. 2014; Battersby 2017; Berger and van Helvoirt 2018). In this sense, the high appreciation particularly of both forms of urban gardening (self-supply and commercial) by local stakeholders is comprehensible.

Apart from some regional differences, the SIA applied also reveals apparently ‘chain-inherent’ effects (‘basic principles’) of the chain types. The different SFSC perform very well in the social dimension and in nuances also in the economic dimension across all case study cities, but certain chain types show typical weaknesses (e.g. transportation efficiency, eco-efficiency or profitability). This might be inherent to specific scales and organisational structures, which several statements from reviews and empirical studies suggest (Brunori et al. 2016; Mundler and Lauhgrea 2016; Malak-Rawlikowska et al. 2019; Vittersø et al. 2019). Longer chains seem to generate lower environmental impacts per unit (e.g. measured in food miles or transportation distance), which is worst in the case of on-farm sales (Malak-Rawlikowska 2019). This raises questions about economies and ecologies of scale of SFSC, but it needs to be discussed together with positive environmental impacts, for example, agro-biodiversity, landscape diversity and the provision of ecosystem services (Mundler and Laughrea 2016), where is also a high societal demand. To sum up, we share the estimation by other researchers that the different global and SFSC types have their specific strengths and weaknesses and differ in their potential contribution towards sustainable food systems (Brunori et al. 2016; Vittersø et al. 2019).

The SIA-SFSC can be seen as pilot study testing a different methodological approach for the sustainability assessment of SFSC based on food chain actors and stakeholder judgements. The SIA is neither a top-down nor bottom-up approach (Binder 2010) but can be classified as a ‘down up’ in the sense that it was developed by experts from various disciplines, and tested and applied by practitioners with reflection on the whole procedure. Although the input data for the SIA-SFSC as a rapid assessment tool is qualitative and the assessment is rather subjective (Marchand et al. 2014), the results are comparable with those from other tools and methods applied, especially in full sustainability assessments (e.g. Malak-Rawlikowska et al. 2019) or found in reviews (e.g. Benedek et al. 2020). This might help overcome the general assumption that SFSC are more sustainable and show the need for more differentiated reflections. This is also reported from London when comparing SIA assessments with on-site consumer and producer interviews in a community-led local market (Schmutz et al. 2017).

City-regional policies and strategies are mostly based on normatively set visions. Having pointed to the fact that the assessments are carried out from a subjective perspective, we refer to Kemp and Martens, who argue “that sustainable development is an inherently subjective concept and for this reason requires deliberative forms of governance and assessment” (2007, p. 5), which also includes integrated, participatory assessments.

Similarly, single SFSC businesses and initiatives develop their business models. Here, sustainability plays a role from an economic perspective, but also incorporates subjective value orientations. Finally, we can assume changes in the decision-making behaviour towards sustainability being determined by behavioural factors (knowledge, social aspects, individual disposition), as described for the adoption of sustainable farming practices (Dessart et al. 2019). Additionally, Weltin et al. (2021) found that the shift to SFSCs, specifically the adoption of regional marketing by farmers, depends strongly on farmers’ individual attitudes towards economic and environmental sustainability.

Any SIA evaluation utilising the means of the tool presented here is, thus, not understood or intended to be an objective assessment. The actual value of the tool lies in providing a common and scalable basis of clear, relevant and distinguishable criteria for comparisons among city region SFSC or tracking developments of single SFSC. We are also aware that the qualitative estimations about possible impacts represent the perceptions and assumptions of (local) experts and are connected with ‘uncertainties’ depending on various factors, including farm scale, product, location and chain organisation. Nonetheless, “the assumptions are highly valuable, because they are grounded in the practical realities of trying to build viable sustainable food systems, which deliver a range of public and private goods in a particular social and spatial context” (Schmutz et al. 2017, pp. 3–4). We further argue that insights into the perceptions and knowledge of different food chain stakeholders is essential for food system transitions and can be an element of the “reflexive governance of food chains” (Brunori et al. 2016) in the established SFSC and for the growing number of new food initiatives, food hubs (Barham et al. 2012; Fischer et al. 2015) and organic supermarkets.

However, the SIA-SFSC was not tested for all chain types and in all city regions. Results are available across the four case studies only for vegetable chains. They show some differences, which can be explained by the use of different methods (survey vs. workshop, group discussion), the assessment situation (anonym and individual vs. public and collective estimations), previous knowledge and professional background of the participants. This was also the case in the online survey and regarding regional experts in the SIA workshops. In addition to this, it might be the case that the regional context and the actual performance of the chains studied have influenced the outcome of the SIA in the case study regions, which means the tool is sensitive to the regional settings. Nonetheless, more attention should be paid in future studies to the regional socio-economic situation and the importance of the diverse food chains in the food system investigated (Vittersø et al. 2019).

As we see the field of application in the area of city-regional assessments, the sensitivity regarding regional settings is not a disadvantage and does not limit the universal usability for the evaluation of local initiatives (and possibly benchmarking). The necessary background knowledge for the classification of possible structural, actor- and organisation-related differences is also considered as part of an urban-regional assessment, as is the discussion of the comparative relevance of criteria for the intended strategy and expected contribution of different SFSC profiles within the context of their specific regional settings.

Further research and development is required in order to identify (practical) solutions or design innovations of all kinds (social, technical, organisational) that can improve the sustainability of SFSC. Another relevant question in the field investigated is related to the up-scaling of SFSC or local food chains and their potential sustainability impacts (Eakin et al. 2017). In addition, more attention should be paid to methodological research and comparisons. To the best of our knowledge, there are no reviews or meta-analyses that assess and compare the coverage and quality of results of different methodological approaches. We estimate that research and practice could benefit if the strengths, weaknesses and trade-offs from the diverse local food chains were assessed more systematically. This could contribute to the development of evidence-based theories about the sustainability of SFSC (Tregear 2011; Michel-Villareal et al. 2019).

## Conclusion

We developed a rapid assessment tool utilising the SIA-SFSC which is applicable for practitioners (food chain actors, planners, policymakers) and scientists. Local stakeholders were partly involved in the tool design and testing (indicator, food chain types) and fully in the tool application.

The SIA-SFSC brings a new facet into the existing food chain studies by the assessment of a common set of sustainability aspects for different food chain types, approved and applied by scientific experts at a European level (without regional context) and regional stakeholders and practitioners in case studies.

The advantages of our rapid assessment tool are the reduced number of qualitative sustainability indicators, high transparency, user-friendliness and low requirements for data availability, because the assessment is based on the knowledge and estimation of the (local) food chain actors or scientists. In the face of the rapid growing number of urban food polices and food initiatives around the world, such tools gain new relevance. However, there are also trade-offs when choosing rapid assessment tools such as SIA-SFSC. If higher data accuracy and objectivity is required for policy or enterprise decisions, we would recommend choosing a full assessment tool or combining rapid and full assessment tools.

The findings from our study and from the literature demonstrate the value of differentiating chain types and cross-regional comparisons in the impact assessment of food chains. This provides a good basis for guidance and decision support for food initiatives and policymakers who aim to develop UFP and the shape of urban food systems. The weaknesses identified in terms of ecological and economic impacts give an indication of innovation potentials, whereas the SIA-SFSC can then be used to assess the sustainability of innovations such as food hubs or micro-logistics for local small and medium-sized food producers.

The differences in the results between scientific experts (online survey) and local stakeholders as well as across the case study regions highlight the importance of regional situation adjusted strategies and solutions to SFSC innovation as well as well-defined target systems and indicator sets that were ideally co-developed with local stakeholders. The SIA-SFSC tool can support the profiling of different food chain types towards innovation goals in terms of impact areas which are specific to food chain innovation. Furthermore, the performance of the chain types in specific impact areas also gives some indication regarding how the food topic can be linked with other urban policy targets such as biodiversity or climate protection and where synergies between different sustainability targets can be achieved in the urban setting.

We intend to bridge the gap between the international dimensions of food policy, trade and consumption, on the one hand, and the regional reality of local actors and consumers, on the other hand, by offering a tool for bottom-up processes and specifically for food chain types with closer consumer-producer interaction. The tool also fills the gap between product- or farm-related assessment tools (such as SAFA or LCA) and frameworks that assess the whole food system (local food system assessment) or evaluate the impact of UFP (FAO 2019).

We expect that participatory elements in sustainability food chain and food system assessments will play a greater role in the future in the context of bottom-up driven UFP and questions of food democracy and sovereignty. Although our tool was applied in a participatory manner, the development and indicator selection was mainly science-driven. Nonetheless, participatory indicator development is also conceivable and the tool can be adapted to new concerns arising in society (such as Covid-19).

We found the tool useful for framing the dialogue between food chain actors, consumers and policy because it makes benefits and trade-offs of the chain types operating in an urban–rural context more visible und communicable. The findings of our study and the application of the SIA framework in different regional settings can improve our understanding about the relationship of different food chain types and sustainability.

## Supplementary Information

Below is the link to the electronic supplementary material.

Supplementary file1 (DOCX 32 KB)

Supplementary file2 (DOCX 1386 KB)

## Data Availability

The aggregate data that support the findings of this study are available in the supplementary information of this paper. The original data can be provided on request.

## Abbreviations

AFN: Alternative food networks
CSA: Community supported agriculture
FSA: Full sustainability assessment
LCA: Life cycle assessment
PDO: Protected designation of origin
PGI: Protected geographical indication
RSA: Rapid sustainability assessment
SFSC: Short food supply chains
SIA: Sustainability impact assessment
UFP: Urban food policies
