# Supplementary material for: Sustainability assessment of short food supply chains (SFSC): developing and testing a rapid assessment tool in one African and three European city regions
Source: Agric Human Values. 2022 Feb 24;39(3):885–904. doi: 10.1007/s10460-021-10288-w (PMC8868038; doi:10.1007/s10460-021-10288-w)
Supplement: Supplementary file 1 — Supplementary file1 (DOCX 32 KB) [file 10460_2021_10288_MOESM1_ESM.docx]

Supplementary information part 1: review of assessment tools and frameworks in the agri-food sector

In the following table we give a short overview about assessment tools that address three or more dimensions of sustainability and allow the evaluation of economic, environmental and social aspects in food chains or food systems. We also indicate, based on our own estimations, whether they are rather full or rapid assessment tools (Marchand et al. 2014). The approaches and tools presented were chosen in order to exemplify the broad variety of methods and scopes and the table does not claim to be complete.

**Table 5 Examples of integrated (indicator-based) sustainability assessment tools and frameworks**

| **Name of the frameworks and tools for Sustainability Assessment** | **Original aim of the tool developer /purpose of the tool** | **Target groups** | **Scope (entity) and level of assessment of the tool or its application (empirical study)** | **Method of evaluation and data gathering (data sources)** |
| --- | --- | --- | --- | --- |
| **Benchmarking framework, (integrated) sustainability index***  ………………………….  **Key references:**  Yakovleva et al. 2010, 2012 | Sustainability performance of an organisation (enterprise) and across the supply chain  Assess long-term organisational viability and competitiveness and sustainability of the whole supply chain | Investors, policymakers and other stakeholders | Enterprises along the food chain at national scale (UK)  45 indicators: nine sustainability indicators for each of the five stages in the food chain (agriculture, food processing, food wholesale, food retail, food catering) | Analytical Network Process  Sustainability index  quantitative statistical data combined with expert opinion |
| **Stakeholder Priority and Responsibilities Matrix and other Corporate Social Responsibility (CSR) schemes*****  ………………………….  **Key references:**  DEFRA 2002,  Smith 2008,  Caracciolo et al. 2011 | Support joint decision-making and co-operation of stakeholders within the same supply chain and outside  Identify suitable solutions and priorities (sustainability objectives)  Part of risk assessment and CSR | All actors along the same food supply chain (food business, consumers), institutions, research | Local food chain actors perspective/responsibilities (actors within the chain, outside the chain)  “represents synthetically the relationships between actors and sustainability targets” (Caracciolo et al. 2011) | Qualitative and quantitative data from three sources: semi-structured interviews, secondary data from official statistics and document analysis |
| **Sustainability Assessment of Food and Agriculture Systems SAFA-Guidelines***  ………………………….  **Key references:**  FAO 2013,  Schader et al. 2012,  Malak-Rawlikowska et al. 2019 | Shared and understandable way to communicate about sustainability in agriculture  Assess trade-offs and synergies between all dimensions of sustainability, *“seeks to harmonize sustainability approaches within the food value chain, as well as furthering good practices*” (FAO 2013: V) | Food and agricultural enterprises (companies, small-scale producers), organisations, governments | Indicators for food and agriculture enterprises (individuals or associations) | Type of information: qualitative based on perceptions |
| **Sustainability Assessment of Farming and the Environment (SAFE framework)******  ………………………….  **Key references:**  Sauvenier et al. 2005 | Tool of decision-making in agriculture | Farmers, decision  makers, researchers | Set of core indicators for agricultural systems (many related to functions of agro-ecosystems)  At three spatial levels: (1) parcel, (2) farm or (3) watershed for surface water-related issues, landscape/ecosystem for some soil- and biodiversity- related issues, and administrative units (region, state) for some environmental and socio-economic issues. | Expert-based scoring systems  Data monitoring of four farms (field data and questionnaires)  Applied for ten case studies |
| **MOTIFS (Monitoring Tool for Integrated Farm Sustainability)*****  ………………………….  **Key references:**  Meul et al. 2008,  Triste et al. 2014 | To provide a user-friendly and communicative monitoring tool that allows the measurement of progress toward integrated sustainability at dairy farms | Farmers and farm advisors | Farm level | Combination of expert information and detailed farm data  Scan of readily, available data, extended questionnaires on farm evaluations by experts |
| **Public Goods (PG) Tool***  ………………………….  **Key references:**  Gerrard et al. 2011, 2012 | To provide a tool to assess the public goods provided by a farm  Benchmarking and learning tool for (organic)farmers | Farmer, society | Farm level: (organic) farms | Combination of available accountancy data, cropping/livestock records, and farmer knowledge |
| **Social Return on Investment Methodology***  ………………………….  **Key references**  SROI Network 2012,  Schmutz et al. 2014,  Foresi et al. 2016 | Social value generated by an entire organisation or one specific aspect of the organisation’s work. | Stakeholder involved in (social) enterprises and organisations | Organisations or projects (e.g. dealing with food production, such as urban gardens)  Deals with social, environmental and health benefits and gives them a financial value  Values the full range of benefits to all stakeholders | Case studies and qualitative, quantitative and financial information |
| **Slow Food Criteria for Sustainable Agri-Food Systems (SAFS)***  ………………………….  **Key references:**  Peano et al. 2015 | Evaluation of products applied at the Slow Food Foundation | Producer associations, Slow Food Presidium | Five-dimensional framework (quality, economic, social, environmental and cultural dimensions)  Indicator-based approach with 41 indicators | Quantitative (numeric range)  Qualitative (presence or absence, different type)  Three case studies at two different points of time  Document analysis, producers’ data and semi-structured interviews |
| **Community-based food system assessment (CFSA)****  ………………………….  **Key references**  Ross and Simce 2008, Miewald 2009 | Create awareness and support informed decision making etc.  Description of the current state and vision building  Development of concrete strategies and programmes, set priorities and identify partners and resources, etc. | Include full range of food system actors (producers, distributers and service providers, and applied and academic research | Food system of a community (neighbourhood, city or region)  Including health, socio-cultural and food policy indicators | Participatory and collaborative process  Indicator-based  either secondary data, information that has already been collected (e.g. census data) or primary data (e.g. data gathering using surveys or interviews) |
| **Sustainability Assessment Tool for Urban Food Governance (based on SAFA)****  ………………………….  **Key references**  Landert et al. 2017 | Assessment of degree of (policy) goal achievement | Stakeholders from public administration, retail, farmers, urban gardening associations, etc. | Urban food systems  Environmental, economic, social well-being plus good governance  Mainly food producers perspective, despite food sovereignty, public health  21 themes with 58 sub-themes (not all were applied in the empirical study) | Multi-criteria analysis with weighted indicators  Document analysis, interviews with different food system actors |
| **Milan Urban Food Policy Pact Monitoring Framework (MUFFP-MF, FAO 2019)*****  **………………………….**  **Key references**  FAO 2019 | To facilitate the design of policies and initiatives  The Framework has been designed to support cities and sub-national officers responsible for designing food systems policies, projects and investments  “The framework has not been designed to compare cities and establish global ranking systems” (FAO 2019, p. 6) | Primarily municipal governments, development  professionals and food practitioner | Urban food systems  44 indicators, set of outcome areas, set of recommend actions | Quantitative calculation resulting from data collection and analysis  Qualitative  self-assessment of the existence and effectiveness of policies, plans, interventions, initiatives |

Own compilation based on Ness et al. (2007); UNEP (2009); Binder (2010); Yakovleva et al. (2010); Singh et al. (2012); Marchand et al. (2014); Zasada et al. (2014); Foresi et al. (2016); de Olde et al. (2016); FAO (2019); Santini et al. (2019)

FSA: Full assessment tool*, RSA: Rapid assessment tool**, rather mixed***, rather a framework or a methodology than a tool****

**Literature cited in supplementary information**

Binder, C.R., G. Feola, and J.K. Steinberger. 2010. Considering the normative, systemic and procedural dimensions in indicator-based sustainability assessments in agriculture. *Environmental Impact Assessment Review* 30(2): 71–81. doi: 10.1016/j.eiar.2009.06.002

Caracciolo, F., M.T. Gorgitano, P. Lombardi, G. Sannino, and F. Verneau. 2011. Responsibility and sustainability in a food chain: A priority matrix analysis. *International Journal on Food System Dynamics* 2(3): 292–304. doi: 10.18461/ijfsd.v2i3.238

De Olde, E.M., F.W. Oudshoorn, C.A.G. Sørensen, E.A.M. Bokkers, and I.J.M. de Boer. 2016. Assessing sustainability at farm-level: Lessons learned from a comparison of tools in practice. *Ecological Indicators* 66: 391–404. doi: 10.1016/j.ecolind.2016.01.047

DEFRA – Department for Environment, Food and Rural Affairs. 2011. Indicators for a sustainable food system. York, UK. <http://webarchive.nationalarchives.gov.uk/20130125171715/http://www.defra.gov.uk/statistics/files/defra-stats-foodsystemindicators.pdf>. Accessed 25 November 2021.

FAO – Food and Agriculture Organization of the United Nations. 2013. Sustainability assessment of food and agriculture systems. SAFA indicators. Rome. <http://www.fao.org/fileadmin/templates/nr/sustainability_pathways/docs/SAFA_Indicators_final_19122013.pdf>. Accessed 25 November 2021.

FAO – Food and Agriculture Organization of the United Nations. 2019. The Milan urban food policy pact monitoring framework. Rome. http://www.fao.org/3/ca6144en/ca6144en.pdf. Accessed 25 November 2021.

Foresi, L., U. Schmutz, A. Anton, A. Vieweger, M. Bavec, M. Meier, M. Shadid, N. Pena, R. Petrasek, D. Stajnko, T. Vukmanic, J. Landert, and R. Weisshaidinger. 2016. *Sustainability assessment tools for organic greenhouse horticulture*. Wageningen: BioGreenhouse. <https://library.wur.nl/WebQuery/wurpubs/499458>

Gerrard, C., L. Smith, S. Padel, B. Pearce, R. Hitchings, M. Measures, and N. Cooper. 2011. OCIS public goods tool development. Organic Research Centre Report. <https://orgprints.org/18518/2/OCIS_PG_report_April_ORC_2011V1.0.pdf>. Accessed 25 November 2021.

Gerrard, C.L., L.G. Smith, B. Pearce, S. Padel, R. Hitchings, M. Measures, and N. Cooper. 2012. Public goods and farming. In *Farming for food and water security*, ed. E. Lichtfouse, 1–22. Sustainable Agriculture Reviews, vol. 10. Dordrecht: Springer.

Landert, J., C. Schader, H. Moschitz, and M. Stolze. 2017. A holistic sustainability assessment method for urban food system governance. *Sustainability* 9(4): 490. doi: 10.3390/su9040490

Malak-Rawlikowska, A., E. Majewski, A. Wąs, S.O. Borgen, P. Csillag, M. Donati, R. Freeman, V. Hoàng, J.-L. Lecoeur, M.C. Mancini, A. Nguyen, M. Saïdi, B. Tocco, Á. Török, M. Veneziani, G. Vittersø, and P. Wavresky. 2019. Measuring the economic, environmental, and social sustainability of short food supply chains. *Sustainability* 11(15): 4004. doi: 10.3390/su11154004

Marchand, F., L. Debruyne, L. Triste, C. Gerrard, S. Padel, and L. Lauwers. 2014. Key characteristics for tool choice in indicator-based sustainability assessment at farm level. *Ecology and Society* 19(3). doi: 10.5751/ES-06876-190346

Meul, M., S. Passel, F. Nevens, J. Dessein, E. Rogge, A. Mulier, and A. Hauwermeiren. 2008. MOTIFS: A monitoring tool for integrated farm sustainability. *Agronomy for Sustainable Development* 28(2): 321–332. doi: 10.1051/agro:2008001

Miewald, C.. 2009. *Community food system assessment. A companion tool for the guide.* Vancouver. <http://www.bccdc.ca/pop-public-health/Documents/communityfoodsystemassessmentacompaniontoolfortheg.pdf>. Accessed 25 November 2021.

Ness, B., E. Urbel-Piirsalu, S. Anderberg, and L. Olsson. 2007. Categorising tools for sustainability assessment. *Ecological Economics* 60(3): 498–508. doi: 10.1016/j.ecolecon.2006.07.023

Peano, C., N. Tecco, E. Dansero, V. Girgenti, and F. Sottile. 2015. Evaluating the sustainability in complex agri-food systems: The SAEMETH framework. *Sustainability* 7(6): 6721–6741. doi: 10.3390/su7066721

Ross, S., and Z. Simce. 2008. *Community food assessment guide.* Vancouver. <http://www.bccdc.ca/pop-public-health/Documents/communityfoodassessmentguide.pdf>. Accessed 25 November 2021.

Santini, G., J. Carey, and B. Cook. 2019. *MUFPP monitoring framework. development and implementation*. 5th Annual Gathering and Mayors Summit, Montpelier, 7 October 2019. <https://www.milanurbanfoodpolicypact.org/wp-content/uploads/2020/02/FAO-MUFPP-Indicator-framework-Montpellier_9Oct.pdf>. Accessed 25 November 2021.

Sauvenier, X., J. Valckx, N. van Cauwenbergh, E. Wauters, H. Bachev, K. Biala, C. Bielders, V. Brouckaert L. Franchois, V. Garcia-Cidad, S. Goyens, M. Hermy, E. Mathijs, B. Muys, J. Reijnders, M. Vanclooster, B. van der Veken, and A. Peeters. 2005. *Framework for assessing sustainability levels in Belgian agricultural systems – SAFE.* Brussels. https://www.belspo.be/belspo/organisation/Publ/pub_ostc/CPgen/rappCP28leaflet_en.pdf. Accessed 25 November 2021.

Schader, C., J. Grenz, M.S. Meier, and M. Stolze. 2014. Scope and precision of sustainability assessment approaches to food systems. *Ecology and Society* 19(3). doi: 10.5751/ES-06866-190342

Schader, C, M. Stolze, and A. Gattinger. 2012. Environmental performance of organic farming. In *Green technologies in food production and processing*, eds. J.I. Boye and Y. Arcand, 183–210. Food Engineering Series. New York: Springer.

Schmutz, U., P. Courtney, and E. Bos. 2014. *Growing for health and happiness. The social return on investment (SROI) of the master gardener programme*. <https://curve.coventry.ac.uk/open/file/8e9ad7d9-9a62-4eb0-93bb-f4e3ab52d271/1/bos4comb.pdf>. Accessed 25 November 2021.

Singh, R.K., H.R. Murty, S.K. Gupta, and A.K. Dikshit. 2012. An overview of sustainability assessment methodologies. *Ecological Indicators* 15(1): 281–299. doi: 10.1016/j.ecolind.2011.01.007

Smith, B.G. 2008. Developing sustainable food supply chains. *Philosophical transactions of the Royal Society of London. Series B, Biological Sciences* 363(1492): 849–861. doi: 10.1098/rstb.2007.2187

SROI Network. 2012. *A guide to social return on investment*. <https://www.socialvalueuk.org/resources/sroi-guide/> Accessed 24 November 2021.

Triste, L., F. Marchand, L. Debruyne, M. Meul, and L. Lauwers. 2014. Reflection on the development process of a sustainability assessment tool: Learning from a Flemish case. *Ecology and Society* 19(3): 47. doi: 10.5751/ES-06789-190347

UNEP – United Nations Environment Programme. 2009. *Guidelines for social life cycle assessment of products.* Paris. https://www.unep.org/resources/report/guidelines-social-life-cycle-assessment-products. Accessed 25 November 2021.

Yakovleva, N., J. Sarkis, and T. Sloan. 2010. Sustainability indicators for the food supply chain. In *Environmental assessment and management in the food industry: Life cycle assessment and related approaches*, eds. U. Sonesson and J. Berlin, 297–329. Woodhead Publishing series in food science, technology and nutrition, vol. 194. Oxford: WP Woodhead Publ.

Yakovleva, N., J. Sarkis, and T. Sloan. 2012. Sustainable benchmarking of supply chains: the case of the food industry. *International Journal of Production Research* 50(5): 1297–1317. doi: 10.1080/00207543.2011.571926

Zasada, I., A. Doernberg, A. Piorr, M. Pintar, M. Glavan, U. Schmutz, E. Bos, L. Venn, T. Mbatia, R. Simiyu, S. Owour, E.D. van Asselt, D. Wascher, and G. Sali. 2014. *Metropolitan footprint analysis and sustainability impacts assessment of SFC scenarios. FOODMETRES Report D5.1 (Update)*. http://www.planningclimatechange.org/public/file/11.%20FoodMetersD5.1%20.pdf. Accessed 24 November 2021.
