# Supplementary material for: Sustainability assessment of short food supply chains (SFSC): developing and testing a rapid assessment tool in one African and three European city regions
Source: Agric Human Values. 2022 Feb 24;39(3):885–904. doi: 10.1007/s10460-021-10288-w (PMC8868038; doi:10.1007/s10460-021-10288-w)
Supplement: Supplementary file 2 — Supplementary file2 (DOCX 1386 KB) [file 10460_2021_10288_MOESM2_ESM.docx]

Supplementary information part 2: tool testing and additional assessment results

Sampling in the online expert survey

Relevant experts and scholars are identified using systematic search via the literature databases Scopus (Elsevier) and Web of Knowledge (Thompson Reuters). We searched for peer‐reviewed articles published between 2000 and 2013 in the English language, using the search terms listed (Table 6).

**Table 6 Search terms used for the identification of experts**

| **Search terms** | **Number of hits in Scopus database** | **Number of hits in Web of Science database** |
| --- | --- | --- |
| “short food supply” | 28 | 19 |
| “alternative food networks” | 71 | 67 |
| “alternative agro-food networks” | 8 | 5 |
| “direct marketing” AND “agriculture” | 42 | 35 |
| “assessment” AND “food supply chain*” | 92 | 63 |
| “sustainability” AND “food supply chain*” | 72 | 31 |

The expert sample featured the following characteristics: 107 of the experts are male, 71 are female. The experts identified work in 23 different European countries, whereas they originate mainly from the United Kingdom (58), the Netherlands (18) and Italy (18). The experts identified held positions mainly at universities and public research institutes, but also at research units in enterprises and they are experts in the fields of agricultural, environmental, social, economic and life sciences.

**Table 7 Documentation of revised impact areas and indicators**

| **Impact Areas for the pre-test and pilot study (regional workshop Berlin)** | **Kind of revision** | **Revised Impact Areas after pre-test and pilot study** |
| --- | --- | --- |
| 1. **Environment** | | |
| 1.1 Environmental friendly farming system: | Deleted |  |
| 1.2 Efficient resource use (water, energy, nutrient, land) | Kept, but specified to abiotic resources | 1.1 Eco-efficiency in abiotic resource use (land/soil, water, nutrients) |
| 1.3 Animal protection and health | Kept, but renamed | 1.3 Animal protection and welfare |
| 1.4 Ecological preservation and and (agro-) biodiversity | Kept, but renamed | 1.2 Provision of ecological habitats and (agro-)biodiversity |
| 1.5 Reduction of emission and pollution (NOx, Sox etc.) | Deleted |  |
| 1.6 Reduction of transportation distance | Kept | 1.4 Reduction of transportation distance |
|  | New impact area | 1.5 Reduction of packaging |
| 1. **Economy** | | |
| 2.1 Efficient transport from producer to consumer | Kept | 2.4 Transportation efficiency |
| 2.2 Increase income / profits for the producers | Kept, but renamed | 2.2 Income and profitability |
| 2.3 Generating employment along the food chain | Kept | 2.1 Employment along the food chain |
| 2.4 Reduces of food losses and waste along the food chain | Kept | 2.5 Reduction of food loss and waste along the food chain from producer to households |
| 2.5 Improve human health and reduces diet-related diseases | Deleted |  |
| 2.6 Improve rural viability | Kept, but renamed | 2.3 Rural viability and competitiveness |
| 1. **Society/culture** |  |  |
| 3.1 Food safety | Kept, but renamed | 3.1 Food safety and human health |
| 3.2 Food quality | Kept, but specified | 3.2 Food quality (freshness, taste and nutritional value) |
| 3.3 Viability of food traditions and culture | Kept | 3.3 Viability of food traditions and culture |
| 3.4 Transparency and traceability | Kept | 3.4 Transparency and traceability |
| 3.5 Availability of food | Merged under the impact areas “food security” | 3.5 Food security (availability and accessibility) |
| 3.6 Affordability and accessibility of food | Merged under the impact areas “food security” |  |

**Figures 4--18 Comparison between the online expert survey and regional case study workshop results (London, Ljubljana, Nairobi)**

| 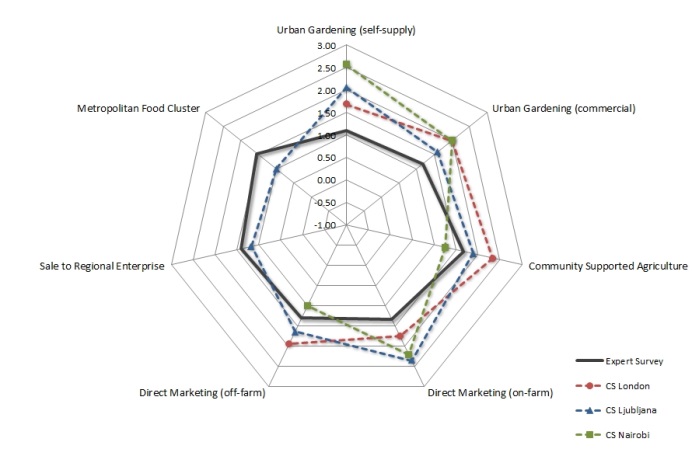 | 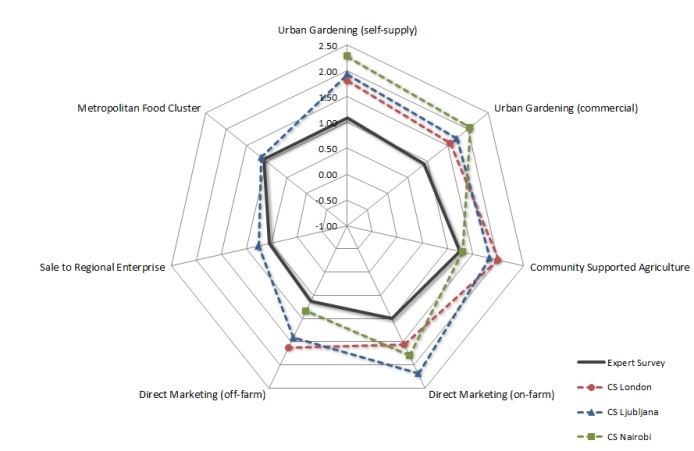 |
| --- | --- |
| **Figure 4 Env 1.1 Eco-efficiency in abiotic resource use (land/soil, water, nutrients)** | **Figure 5 Env 1.2 Provision of ecological habitats and**  **(agro-)biodiversity** |
| 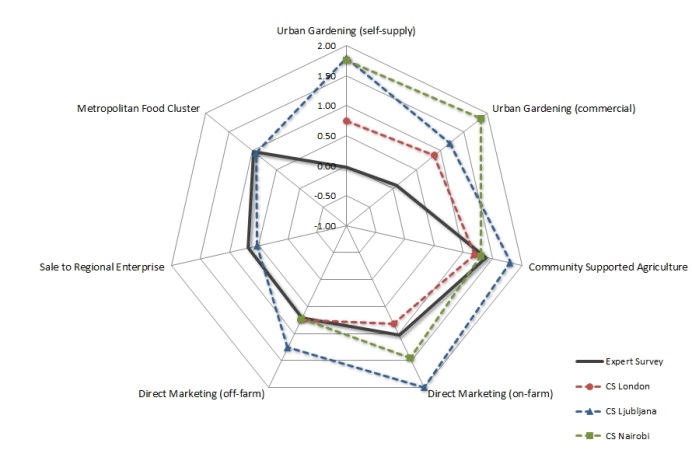 | 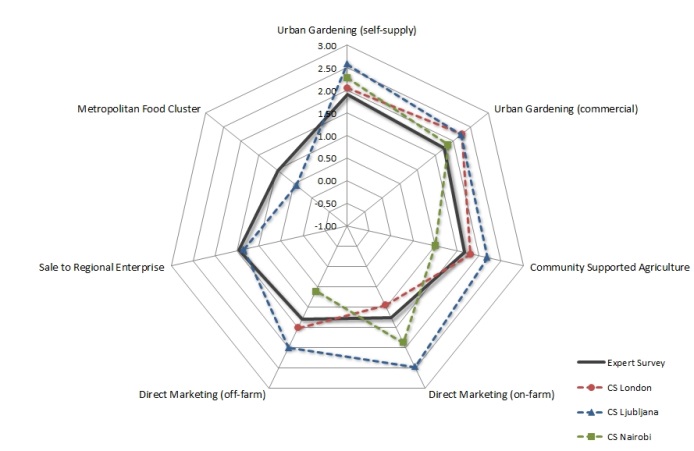 |
| **Figure 6 Env 1.3 Animal protection and welfare** | **Figure 7 Env 1.4 Reduction of transportation distance** |
| 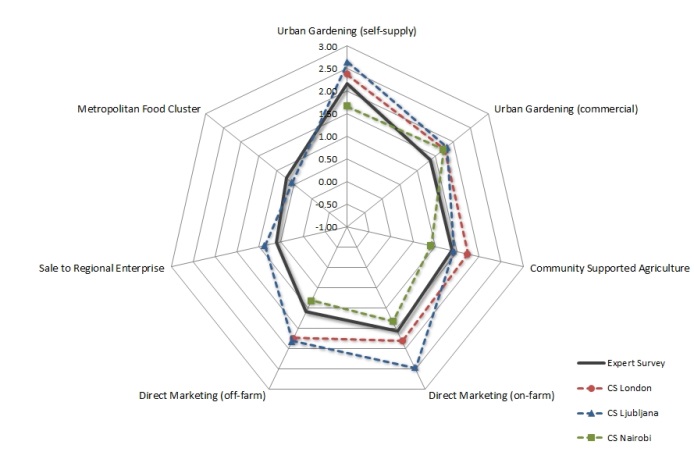 | 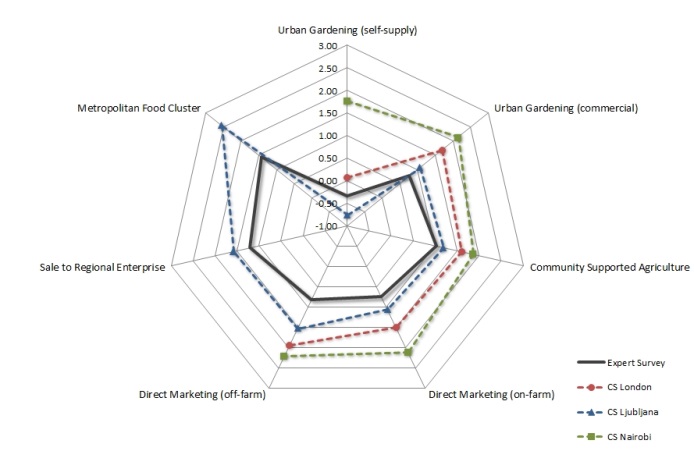 |
| **Figure 8 Env 1.5 Reduction of packaging** | **Figure 9 Eco 2.1 Employment along the food chain** |
| 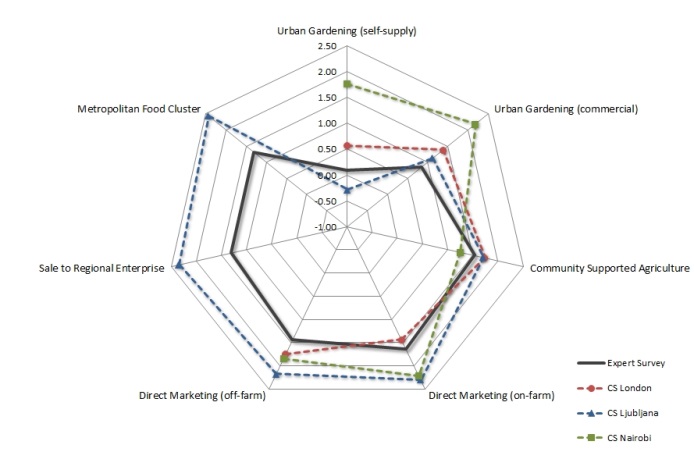 | 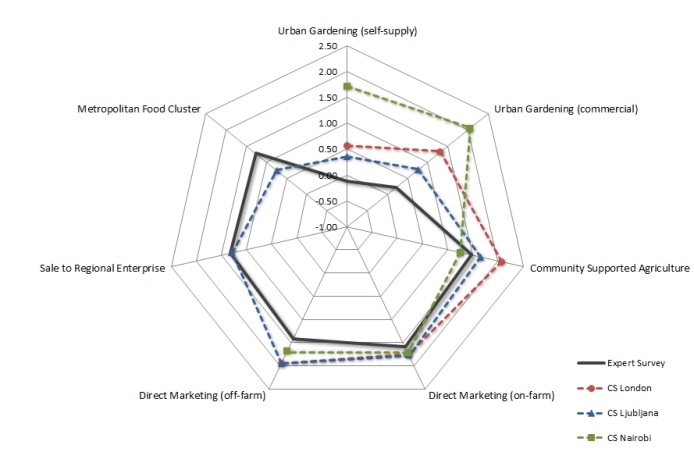 |
| **Figure10 Eco 2.2 Generating income and profitability** | **Figure 11 Eco 2.3 Rural viability and competitiveness** |
| 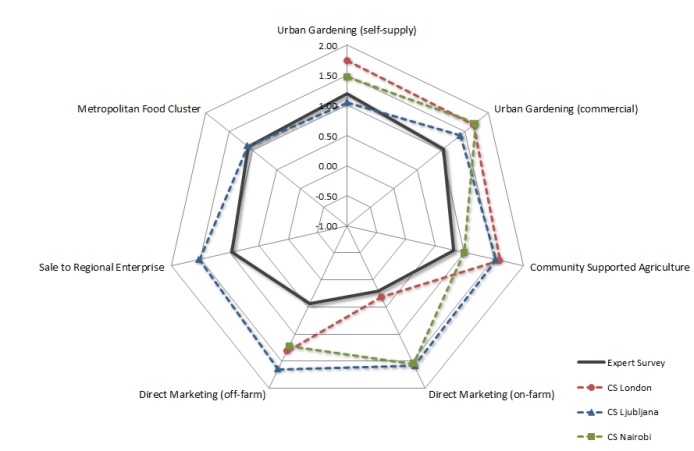 | 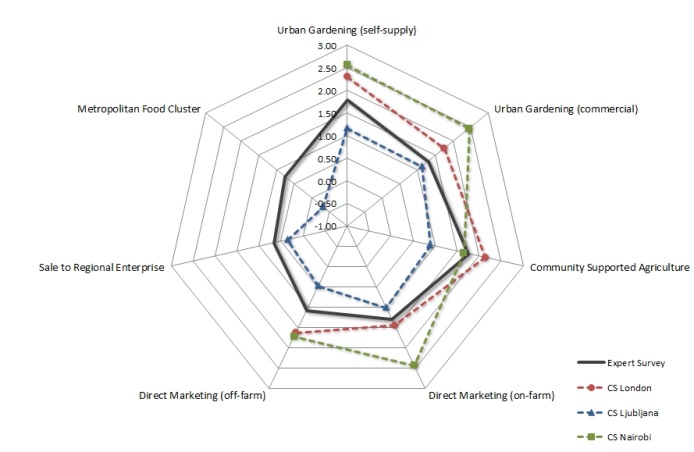 |
| **Figure 12 Eco 2.4 Transportation efficiency** | **Figure 13 Eco 2.5 Reduction of food loss and waste** |
| 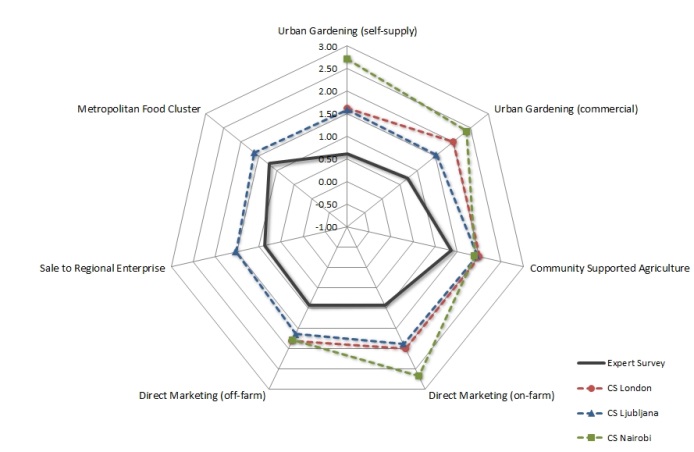 | 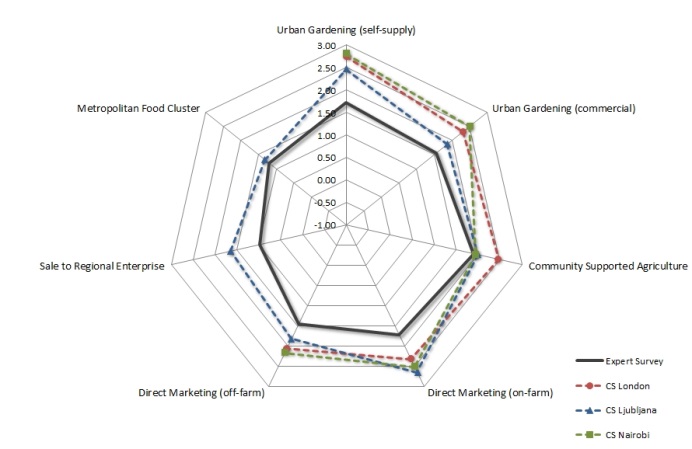 |
| **Figure 14 Soc 3.1 Food safety and human health** | **Figure 15 Soc 3.2 Food quality (freshness, taste, nutritional value)** |
| 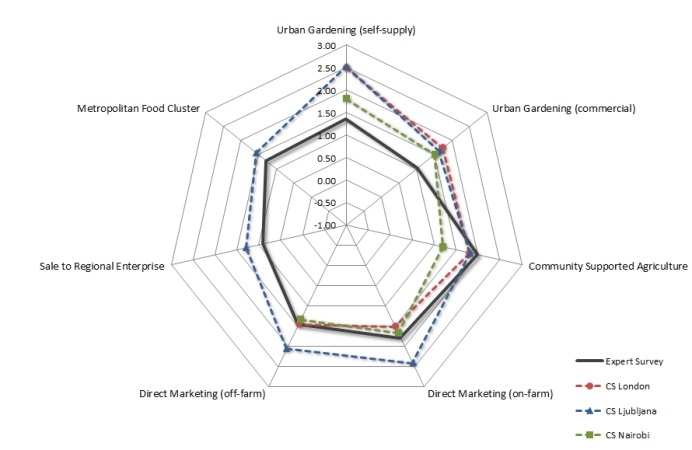 | 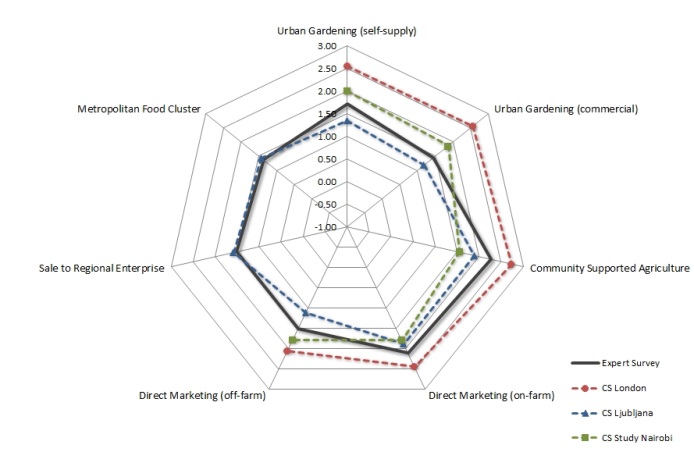 |
| **Figure 16 Soc 3.3 Viability of food traditions and culture** | **Figure 17 Soc 3.4 Transparency and traceability** |
| 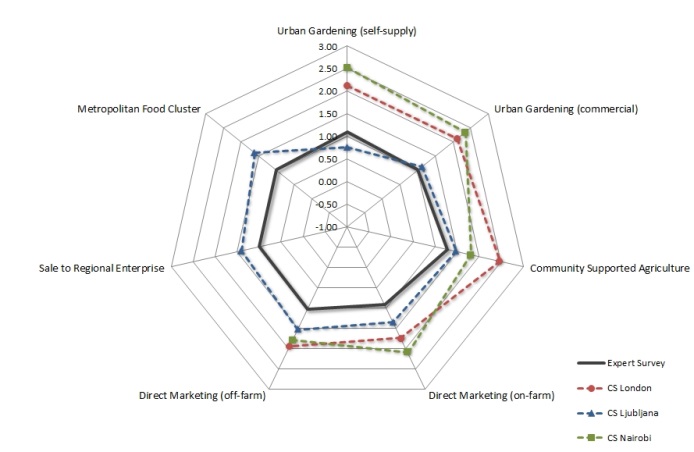 |  |
| **Figure 18 Soc 3.5 Food security (availability and accessibility).** |  |

*Source: Zasada et al. 2014*

**Results from the SIA for other commodity groups**


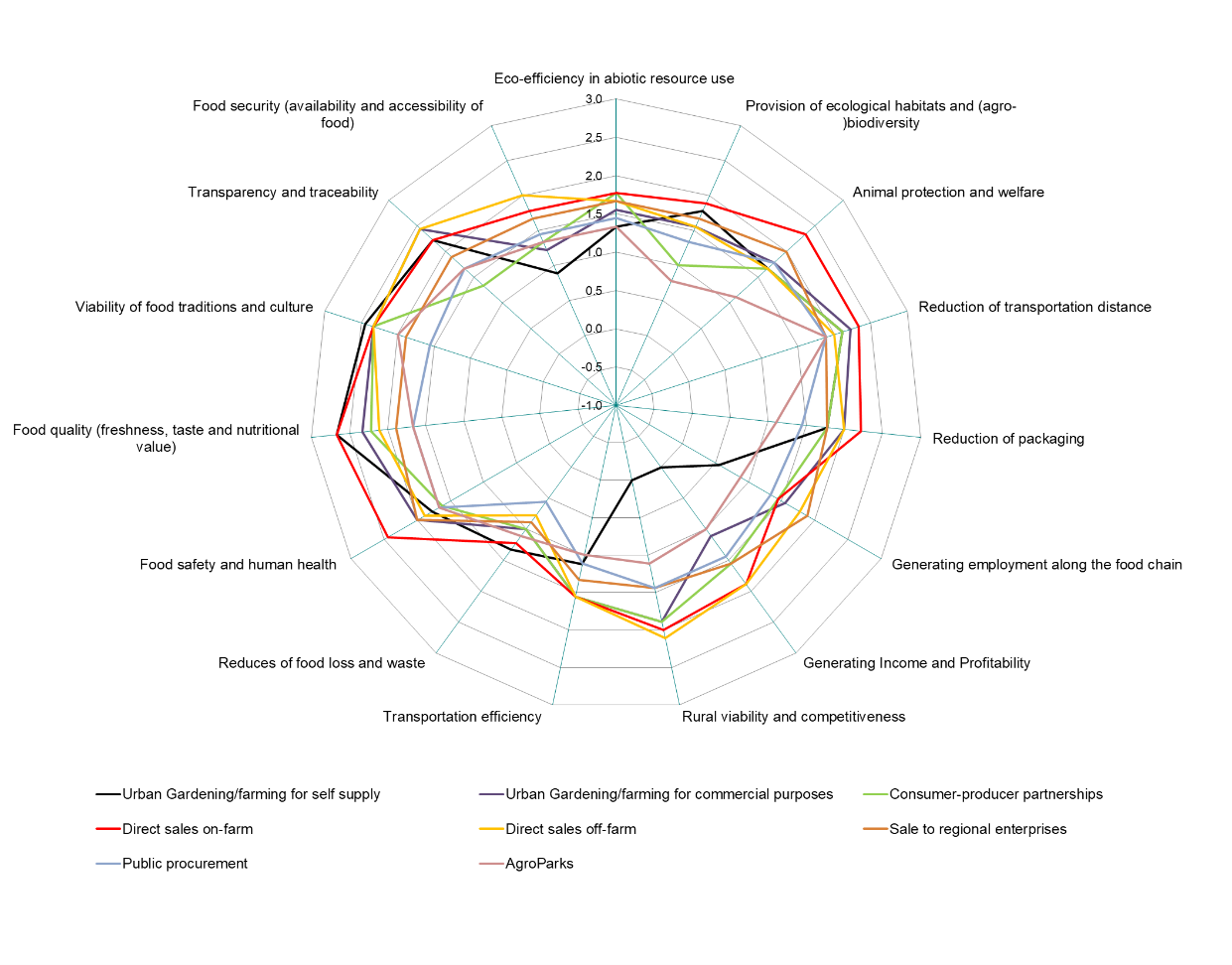


**Figure 19 SIA results for pork meat (Ljubljana, N=9),**

*source: Zasada et al. 2014*


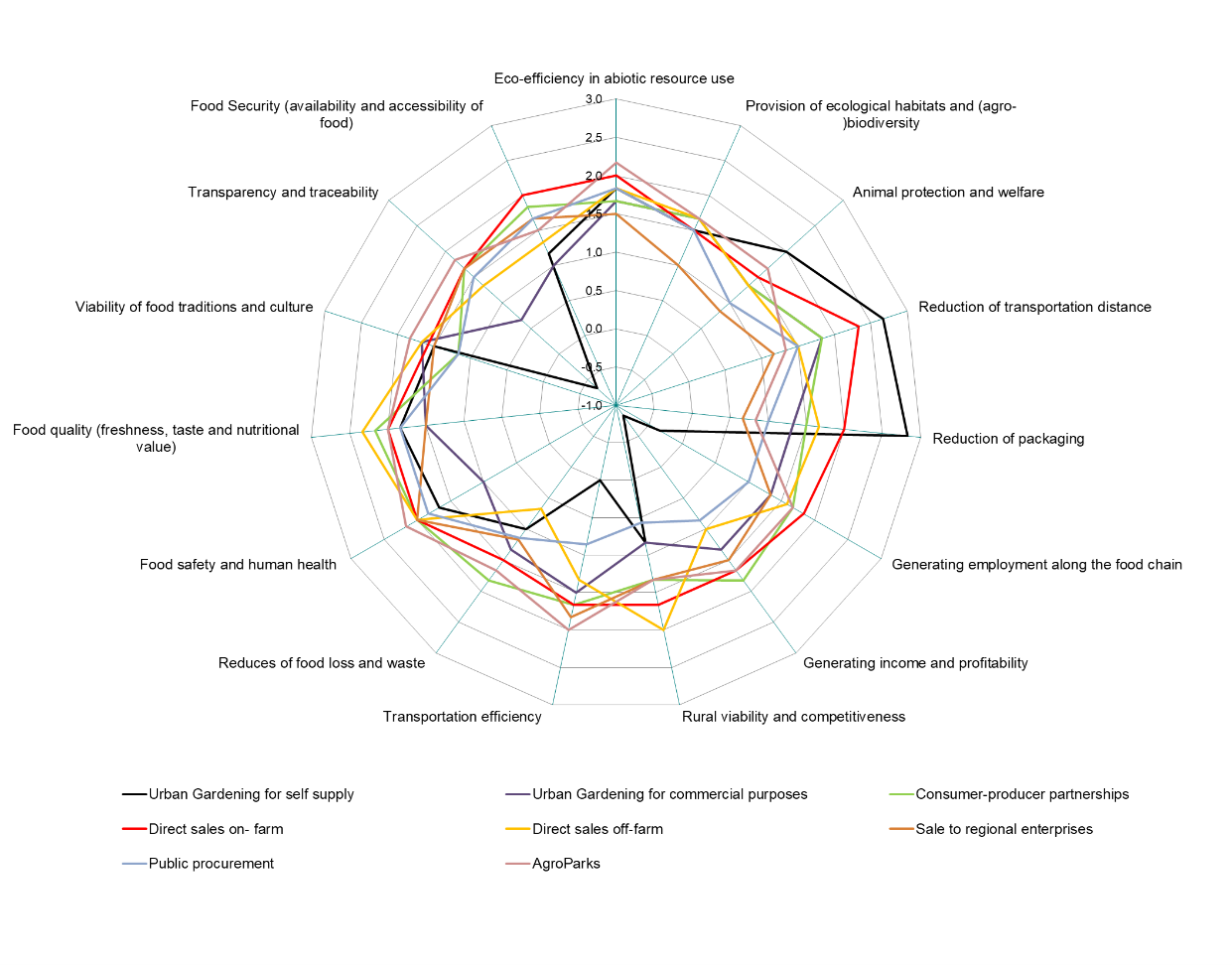


**Figure 20 SIA results for fruits (Ljubljana, N=6)**

*source: Zasada et al. 2014*

**Literature cited**

Zasada, I., A. Doernberg, A. Piorr, M. Pintar, M. Glavan, U. Schmutz, E. Bos, L. Venn, T. Mbatia, R. Simiyu, S. Owour, E.D. van Asselt, D. Wascher, and G. Sali. 2014. *Metropolitan footprint analysis and sustainability impacts assessment of SFC scenarios. FOODMETRES report D5.1 (Update)*. http://www.planningclimatechange.org/public/file/11.%20FoodMetersD5.1%20.pdf. Accessed 24 November 2021.
